# Author Correction: Biological heterogeneity in idiopathic pulmonary arterial hypertension identified through unsupervised transcriptomic profiling of whole blood

**DOI:** 10.1038/s41467-022-33381-y

**Published:** 2022-11-25

**Authors:** Sokratis Kariotis, Emmanuel Jammeh, Emilia M. Swietlik, Josephine A. Pickworth, Christopher J. Rhodes, Pablo Otero, John Wharton, James Iremonger, Mark J. Dunning, Divya Pandya, Thomas S. Mascarenhas, Niamh Errington, A. A. Roger Thompson, Casey E. Romanoski, Franz Rischard, Joe G. N. Garcia, Jason X.-J. Yuan, Tae-Hwi Schwantes An, Ankit A. Desai, Gerry Coghlan, Jim Lordan, Paul A. Corris, Luke S. Howard, Robin Condliffe, David G. Kiely, Colin Church, Joanna Pepke-Zaba, Mark Toshner, Stephen Wort, Stefan Gräf, Nicholas W. Morrell, Martin R. Wilkins, Allan Lawrie, Dennis Wang, UK National PAH Cohort Study Consortium, Marta Bleda, Charaka Hadinnapola, Matthias Haimel, Kate Auckland, Tobias Tilly, Jennifer M. Martin, Katherine Yates, Carmen M. Treacy, Margaret Day, Alan Greenhalgh, Debbie Shipley, Andrew J. Peacock, Val Irvine, Fiona Kennedy, Shahin Moledina, Lynsay MacDonald, Eleni Tamvaki, Anabelle Barnes, Victoria Cookson, Latifa Chentouf, Souad Ali, Shokri Othman, Lavanya Ranganathan, J. Simon R. Gibbs, Rosa DaCosta, Joy Pinguel, Natalie Dormand, Alice Parker, Della Stokes, Dipa Ghedia, Yvonne Tan, Tanaka Ngcozana, Ivy Wanjiku, Gary Polwarth, Rob V. Mackenzie Ross, Jay Suntharalingam, Mark Grover, Ali Kirby, Ali Grove, Katie White, Annette Seatter, Amanda Creaser-Myers, Sara Walker, Stephen Roney, Charles A. Elliot, Athanasios Charalampopoulos, Ian Sabroe, Abdul Hameed, Iain Armstrong, Neil Hamilton, Alex M. K. Rothman, Andrew J. Swift, James M. Wild, Florent Soubrier, Mélanie Eyries, Marc Humbert, David Montani, Barbara Girerd, Laura Scelsi, Stefano Ghio, Henning Gall, Ardi Ghofrani, Harm J. Bogaard, Anton Vonk Noordegraaf, Arjan C. Houweling, Anna Huis in’t Veld, Gwen Schotte, Richard C. Trembath

**Affiliations:** 1 Department of Neuroscience, University of Sheffield, Sheffield, UK; 2 Department of Infection, Immunity & Cardiovascular Disease, University of Sheffield, Sheffield, UK; 3 Department of Medicine, University of Cambridge, Cambridge, UK; 4 Royal Papworth Hospital, Cambridge, UK; 5 National Heart and Lung Institute, Imperial College London, London, UK; 6 Sheffield Pulmonary Vascular Disease Unit, Royal Hallamshire Hospital, Sheffield, UK; 7 Department of Cellular and Molecular Medicine, University of Arizona, Tucson, AZ USA; 8 Department of Medicine, University of California, San Diego, La Jolla, CA USA; 9 Department of Medicine, Indiana University, Indianapolis, IN USA; 10 Royal Free Hospital, University College London, London, UK; 11 Newcastle University, Newcastle, UK; 12 Insigneo institute for In Silico Medicine, Sheffield, UK; 13 University of Glasgow, Glasgow, UK; 14 Department of Computer Science, University of Sheffield, Sheffield, UK; 15 Singapore Institute for Clinical Sciences, Singapore, Singapore; 16 Freeman Hospital, Newcastle, UK; 17 Golden Jubilee National Hospital, Glasgow, UK; 18 Great Ormond Street Hospital, London, UK; 19 Hammersmith Hospital, London, UK; 20 Royal Brompton Hospital, London, UK; 21 Royal Free Hospital, London, UK; 22 Royal United Bath Hospitals, Bath, UK; 23 Sheffield NIHR Clinical Research Facility, Royal Hallamshire Hospital, Sheffield, UK; 24 Département de génétique, hôpital Pitié-Salpêtrière, Assistance Publique-Hôpitaux de Paris, and UMR_S 1166-ICAN, INSERM, UPMC Sorbonne Universités, Paris, France; 25 Université Paris-Sud, Faculté de Médecine, Université Paris-Saclay, AP-HP, Centre de référence de l’hypertension pulmonaire sévère, INSERM UMR_S 999, Hôpital Bicêtre, Le Kremlin-Bicêtre, France; 26 San Matteo, Pavia, Italy; 27 University of Giessen, Giessen, Germany; 28 VU University Medical Center, Amsterdam, The Netherlands; 29 Health and Life Sciences, King’s College, London, UK

**Keywords:** Classification and taxonomy, Functional clustering, Genomic analysis, Cardiovascular diseases

Correction to: *Nature Communications* 10.1038/s41467-021-27326-0, published online 07 December 2021

The original version of this Article omitted Richard C Trembath from the UK National PAH Cohort Study consortium from Health and Life Sciences, King’s College London. This has been corrected in both the PDF and HTML versions of the Article.

