# Supplementary material for: Biological heterogeneity in idiopathic pulmonary arterial hypertension identified through unsupervised transcriptomic profiling of whole blood
Source: Nat Commun. 2021 Dec 7;12:7104. doi: 10.1038/s41467-021-27326-0 (PMC8651638; doi:10.1038/s41467-021-27326-0)
Supplement: Supplementary file 1 — Supplementary Info [file 41467_2021_27326_MOESM1_ESM.pdf]

Heterogeneity of Idiopathic pulmonary arterial hypertension revealed through unsupervised transcriptomic profiling of whole blood

Kariotis *et al*

Supplementary material

Supplementary methods

Cohort population and study design

This expression based study utilizes the UK national H/IPAH cohort including all patients for which idiopathic and heritable PAH was identified in one of the National Centres for Pulmonary Hypertension within the UK, Golden Jubilee National Hospital (n=32), Imperial College Healthcare NHS Trust (n=119), Newcastle Pct (n=31), Papworth Hospital NHS Foundation Trust (n=68), Royal Brompton And Harefield NHS Foundation Trust (n=33), Royal Free Hampstead NHS Trust (n=20) and Sheffield Teaching Hospitals NHS Foundation Trust (n=63). Full written, informed consent with the local ethical committee was required for clinical data as well as blood sampling with the intent of next generation sequencing. The subsequent whole blood sample analysis is described in [1]. Complete information at **Supplementary Data 2**.

Missingness

Clinical data for RNA sequenced patients was assessed for missingness, and patterns within the missing data, prior to analysis. Diagnosis and cohort visit 1 data were analysed separately, using the R packages VIM and Naniar. When looking at clinically relevant variables, overall missingness was 21.35% and 48.64% across the diagnostic and visit 1 datasets, respectively. When focusing on diagnostic variables, the highest percentage of missing data were present in the following variables: BNP (75.77%), left atrial size (73.54%), Troponin (72.42%), NT-proBNP (71.31%), right atrial area (71.03%). High levels of missing data for BNP and NT-proBNP are due to centres carrying out only one of these tests to assess BNP levels. For the other variables, echocardiography is not required for diagnosis and the clinical blood tests chosen are centre dependent. When focusing on cohort visit 1, the highest percentages of missingness can be seen in Troponin (96.94%), jugular venous pressure (89.42%), left atrial size (88.86%), total lung capacity (86.91%) and SvO2 (86.91%).

When looking at variables with the lowest levels of missingness, age (diagnosis) (0.28%), history of syncope (0.28%) , ankle swelling (0.56%) and ascites (0.56%), and weight (2.22%), rank lowest amongst diagnostic data. In comparison, for visit 1, the following variables have the lowest percentages of missingness: functional class (0.84%), ankle swelling (1.11%) and ascites (1.11%), renal sodium (5.01%) and renal urea (5.01%). These variables are recorded routinely when joining the cohort study (for diagnostic variables) or routinely checked as part of clinical examinations.

When analysing missingness per centre, diagnostic data had lower levels of missingness than visit 1. This is unsurprising, as the diagnostic event is more comprehensive than subsequent visits. For the diagnostic dataset, Glasgow had the highest rate of missingness at 36.20% compared to Imperial and Hammersmith which had

the lowest percentage missingness at 19.10%. For cohort visit 1, Glasgow again showed the highest rate of missingness at 71.96%, while Papworth had the lowest at 39.10%. Imperial and Hammersmith accounted for 33.43% of patients in the study, while the other centres contributed between 5.57% and 18.10% of participants, thus inflating missingness statistics. Centre specific differences also exist, with Sheffield using the shuttle test to assess exercise performance in the place of 6MWD. Specific values for missingness calculated for classifier variables only can be seen in **Supplementary Figure 12**, with NT-proBNP exhibiting the highest missingness and age at the time of diagnosis with the lowest missingness, for both diagnosis and cohort visit 1 datasets.

Ethnicity

I/HPAH Cohort study collected and coded the self-reported ethnicity information as per The Office of National Statistics: White: A – British, B – Irish, C – Any other White background, Mixed: D – White and Black Caribbean, E – White and Black African, F – White and Asian, G – Any other mixed background, Asian or Asian British: H – Indian, J – Pakistani, K - Bangladeshi, L – Any other Asian background, Black or Black British: M – Caribbean, N – African, P – Any other Black background, Other Ethnic Groups: R – Chinese, S – Any other ethnic group, Z – Not stated.

Sample and gene selection preprocessing

The initial gene expression dataset consisted of 508 samples, both patients and controls. A number of samples had to be filtered out to ensure a high quality, unskewed input sample set for all subsequent clustering runs. Initially, the first occurrences of three samples were removed from the RNA-seq matrix as they were repeated samples from the same visit of the corresponding patient/control with identical gene expression values. Moreover, 11 patients were excluded as their diagnosis of PAH was not of the idiopathic form and an additional eleven as they were diagnosed with a different form of PH (Pulmonary veno-occlusive disease). Finally, 10 relatives of IPAH patients were excluded due to potentially sharing underlying genetic characteristics with the corresponding IPAH samples.

As part of the preprocessing for the clustering two gene filtering steps were implemented. Firstly, only genes that have more than two reads (in a transcript level) in at least 95% of control and patient samples were considered. This step reduces the number of genes from 60,144 (that occurred after the transcript transformation) to 25,966. Additionally, 11 male genes were removed as they were driving all following clusterings forming subgroups composed entirely by male or female samples.

After selecting for genes and samples a patient gene expression dataset of 385 samples and 25955 genes was generated. The dataset was used to determine the most fitted clustering algorithm in terms of robustness and partition consistency, estimate the optimal number of subgroups and as input for the gene filtering step.

A large number of clinical variables, 121 from the original clinical file and an additional 760 from Open-Clinica study database, were uniformly measured at the different medical and research centers that provided samples as described in the Cohort / Study design section. The date related clinical entries were updated according to the census date of 30.01.2019. Specifically, the date of sampling (defined as the first visit to the corresponding medical center when the blood sample was taken) was composed of additional data on patient samples and healthy volunteers from Imperial and Sheffield local databases. They were mapped to the sample ids to be used either by the clustering algorithms or the downstream biological analyses. Samples with multiple trials were flagged and technical repeats were excluded from the sample pool. Samples with multiple visits (where blood was collected on different dates) were dated to retain the chronological relation between them as useful longitudinal information for the biological analyses.

Feature selection of genes

Next generation sequencing methods measure the expression of thousands of genes providing a huge number of dimensions (>20,000) per sample. However, diseases are usually regulated by smaller groups of genes rather than thousands, as reviewed for PAH in (Ma and Chung 2017). Therefore, the majority of genes are expected not to contribute to PAH development. Additionally, gene filtering helps in reducing the computational burden of most clustering algorithms also affecting their performance by removing misdirecting noise, as shown in (Rodriguez et al. 2019).

Investigating IPAH subgroups concerns the structure underneath the idiopathic form of the disease and is characterised by the complete lack of sample labels. Standard feature selection methods, used for RNA-sequencing data, can not be used in this case as they require labels, e.g. in (Rodriguez et al. 2019; Wenric and Shemirani 2018) where they use disease and control labelling. Therefore, to investigate disease subgroups we ranked all genes based on the variability of their expression across patient samples, as expression variance indicates interesting gene behaviour in our disease context. Each gene was scored according to its variability across the 385 patients using the var() function from the Stats R package. Subsequently, all 25,955 genes were ranked based on that score. To generate the candidate gene sets for the determination of the most stable one, multiple subsets of the top ranked genes were extracted, each time increasing the size by 100.

Highest stability gene set

In our case of feature selection we needed to select one of the gene sets to base the clustering on. Since there are no known IPAH-related genes in the literature we moved forward with the gene sets (one per pipeline) that generated the subgroups of highest stability. We used the established clusterboot function from the fpc R package. The function assesses the clusterwise stability by resampling the data in a bootstrap approach. Then it computes the Jaccard similarities of the original subgroups to the most similar subgroups in the resampled data. Spectral clustering (kernlab R package), 50 resampling runs, k between 2 and 6 and a seed of 28588 for reproducibility purposes was used. We generated multiple gene sets starting from the top 100 ranked genes and increasing the size of the gene set by 100 until we reach the total number of 25,955 genes.

Clustering algorithm selection

There is a wealth of methods in the unsupervised learning field that are appropriate for certain data types. Most studies employ widely-used methods (e.g. hierarchical clustering) without utilising any kind of selection method that would point towards a certain effective methodology. In this study we aimed to examine a group of diverse algorithms that cover different clustering approaches. Since we lacked labels, and thus a performance measure, we compared the partitioning consistency of the different approaches on the expression data. As good consistency we defined high agreement and low standard deviation calculated between different variations of a clustering algorithm. When two different clustering runs agree on the partitioning of the samples they show robustness since they do not randomly assign samples to subgroups but rather are driven by the underlying structure of the data. (**Supplementary Table 6**) presents the various algorithms used, along with various distance measures and clustering categories they belong to.

The preprocessed RNA-seq dataset and k2,10 were used for the determination of the algorithm agreements. Within each pair of clustering runs the agreement was calculated using the adjusted Rand Index, the corrected-for-chance version of the original Rand index (Rand 1971), which is based on the number of times any pair of

points is partitioned in the same subgroup throughout different clusterings. To calculate the intra-agreement of each clustering algorithm (spectral, k-means, hierarchical) we considered only pairs in which both runs were based on the same algorithm. For those pairs the agreement was averaged across clustering runs and ks. In a similar way, the standard deviation per algorithm was calculated.

Optimal number of subgroups k

Estimating the actual number of subgroups is a key decision for every clustering algorithm and is usually based on field knowledge, specific study decisions or statistical indexes. Due to the lack of prior knowledge about the structure of IPAH we are unable to set the number of subgroups based on literature or biology. Since this is an exploratory study (which does not include a predetermined number of subgroups) we are utilizing various indexes depending on the questions we are addressing.

For the case of the IPAH subgroups, we are using ensemble learning, the process where multiple indexes (or experts) participate in the selection of the optimal value of a machine learning decision [2]. Specifically, since we can not use any class related labels, we estimate the optimal number of subgroups using voting among 15 internal indexes that evaluate the compactness and/or the distance between different subgroups (see Supplementary methods**: Internal Index Voting**).

Determining the optimal number of clusters (k) is an inherently difficult task in unsupervised machine learning as it is always an educated estimation, since we do know the actual number of categories within our data. Indeed some of the 14 used indexes are bound to not work on our data type (RNA-seq) and that is why we used an ensemble/voting method to estimate k (supplementary section **Internal index voting**) since we cannot base our estimation on any one index. The voting result (**Supplementary Data 1**) showed the clear majority of indexes to favour up to 5 clusters, with a preference to 2 clusters. The most important aspect in selecting the number of clusters in a data set is retaining as much information as possible, therefore selecting the highest supported k minimizes information lost. Following that notion, we retained the highest voted k = 5, where we discovered 3 distinct adequate sized clusters and 2 small clusters that, despite their interesting gene expression profiles (**Figure 2A**), were unable to show any statistical significance in follow-up work due to their small size. In **Supplementary Figure 16**, we demonstrate the flow of patients between clusterings along with the cluster sizes and the proportion/count of transferring patients across k. The colored nodes represent our subgroups I, II,III, IV and V. According to the clustering tree, the 3 main subgroups I, II and V remain clustered together when k = 2 (in a 341 sized cluster) and k = 3 (in a 295 sized cluster). This indicates that for $k < 5$ we are missing the information that separates these 3 distinct subgroups. The two smaller subgroups (III and IV) mostly originate from a group of patients (circled in green) that dissociates early on from main subgroups I, II and V implying that these samples show some differences even when less subgroups are requested. The remaining samples that end up in subgroup III have a common parent with subgroup II.

Gene signatures of subgroups

A number of biological analyses are used to explore IPAH sub-structure. For the patients of each subgroup survival (Kaplan-Meier curves), response to vasodilators (IPAH treatment), gender and functional class (Fisher’s exact test) are calculated and compared while measuring significance. Additionally, the difference in the age of diagnosis (one-way anova test) and a number of known PAH-related genes are examined across subgroups. We perform a driver gene discovery analysis (LASSO regression model) which can indicate the most influential genes whose literature-generated annotations are investigated. The results of the patient

clustering are used to draw genetic differences between the two groups. IPAH subgroups are subjected to differential expression analysis and subsequently to pathway analysis in order to interpret the genes' involvement in terms of functionality. The p values of each gene when considering the fold change between subgroups I and II were calculated using a Welch Two Sample t-test on the raw values and presented in the **Supplementary Table 4**. The absolute lower cut off values of fold change (log2 scaled) is 0.28.

Internal index voting

To implement ensemble learning, we used the majority voting rule among 15 internal validation indexes, selecting as the optimal number of subgroups (k) the one voted by the most indexes. The various indexes[6–17], (McClain and Rao 1975) and (Ray and Turi 1999) results can be found in **Supplementary Data 1**. All of the above are based on variations of the same idea, to score a partitioning on how compact each subgroup is and how well the subgroups separate. No index can select the real number of subgroups with perfect accuracy, therefore the "voting of experts" method can provide a safer alternative to using any one index. The preprocessed RNA-seq dataset after the appropriate gene filtering step was used for this work.

Clinical variable associations / classification

Survival analysis using a Kaplan-Meier estimate [18] was undertaken to compare the time until death between patient subgroups, a measure able to overcome issues such as subjects withdrawing from the study or not experiencing death during the course of the study's observations. In this cohort withdrawal etiologies include withdrawal by clinician, transplant, leaving the country, and loss to follow up. As the clinical data for the patients did not include a cause of death, it was decided to limit the duration of the analysis to minimise the inclusion of other causes of death. A duration of 10 years from diagnosis was selected as it is a period during which almost 80% of IPAH deaths occur if conventional therapy is used (Kang et al. 2014), while also being sufficient time to allow for useful statistical analysis. Patients who did not die during this period were considered to be alive for the analysis and had their survival time set to 10 years. The Kaplan-Meier model was created using the survival R package to compare survival time between the subgroups, and subsequently plotted using survminer R package (ggsurvplot function). Cox Regression was undertaken to show any statistically significant survival differences between the subgroups. A Cox model was selected as it has been shown to be a more flexible alternative to parametric methods, and does not require the distribution of survival times to be stated (Bradburn et al. 2003). It was noted that patient survival could be affected by factors other than their subgroup membership. Therefore, survival analysis was repeated using a multivariate Cox regression which included the patients' age at diagnosis, sex, and New York Heart Association (NYHA) functional class. This method allows for adjustment to the impact of these other factors, and shows an estimate of their respective strength of effect.

A frequency table was created for vasoresponders, gender and each functional class within each patient subgroup. Pairwise comparisons were made between the subgroups using Fisher's exact test. This test was performed using the rcompanion R package and a Bonferroni correction was used as multiple comparisons were made.

A comparison of the age at IPAH diagnosis was made between the patient subgroups using a one-way anova test. This test assumes that the observations within each subgroup are normally distributed, and that the data are homoscedastic with equal standard deviation between the subgroups. The normality of the data within each subgroup was confirmed visually by producing a histogram for the age of diagnosis for each subgroup, as well as by plotting a histogram of the residuals for the anova model to ensure that these followed an approximately

normal distribution, and creating a Q-Q plot of the residual values. The homoscedasticity was assessed by plotting the residual values against the fitted value, and by using the car R package to perform a Levene's test.

A regression model was used for feature selection of genes whose expression most significantly drove subgroup membership. The RNA-seq counts were split into a training and testing set with a ratio of 70:30. The glmnet R package uses penalised maximum likelihood in order to fit a regression. The model family was set to "multinomial" as the model was to be used to predict a nominal dependent variable with multiple categories, subgroup membership, given gene expression data. As an additional parameter, the type.multinomial was set to "grouped", meaning that multinomial coefficients for a variable were included or excluded together. This also demonstrated an increase in model prediction accuracy during initial testing of models. Ridge, elastic-net, and lasso models were created using the training set, their parameters optimised by cross-validation (cv.glmnet function), and then used to predict the subgroup membership of samples in the test data-set. The models run on the entire data-set to produce coefficients for each gene relating to each subgroup. The elastic-net regression model was preferred based on its ability to select strongly correlated variables in or out together, a useful feature when dealing with genes which may be correlated due to sharing a biological pathway (Zou and Hastie 2005). From the regression results heat maps were produced showing the coefficients for genes in each subgroup. Genes in the top 5% for largest coefficients were selected for further investigation to identify common pathways or functions. It was decided to investigate genes with both positive and negative coefficients, as genes with decreased gene expression which drove subgroup membership are still of biological interest.

The pathfinder R package was used to demonstrate enriched pathways between subgroups. Genes with an absolute LFC in the highest 10% for their subgroup, and with an adjusted p value ≤ 0.05, were inputted to the package.

Identifying clinical signatures of RNA subgroups

The following clinical variables were used during the clinical signature pipeline: age_diagnosis, sex, diagnosis, drug_exposure, bmi, functional_class, 6 minute walking distance, oxygen saturation (pre), oxygen saturation (post), mRAP, mPAP, mPAWP, cardiac output, SvO2, vasoresponse (lenient), vasoresponse (stringent), FEV1, FVC, TLC, KCO, right Atrial Area, Right Ventricle, Tricuspid Apse, emphysema Category, Fibrosis Category, Thromboembolic disease, NT-proBNP, BNP, Urate, inflammation CRP, haem. HB, haem. WBC, haem. Platelets, Renal Sodium, Renal Potassium, Renal Urea, Renal Creatinine, Metabolic Syndrome, Comorbidity HHT, Comorbidity epistaxis, Comorbidity bleed, Comorbidity AVM, Comorbidity Cirrhosis, Comorbidity Hepatitis, Comorbidity PPH, Comorbidity DM1, Comorbidity DM2, Comorbidity Hypothyroidism, Comorbidity SLE, Comorbidity SS, Comorbidity Ankylosing Spondylitis, Comorbidity Sjogren, Comorbidity UCTD, Comorbidity Necrotising Vasculopathies, Comorbidity Overlap Syndrome, Comorbidity Polymyalgia Rheumatica, Comorbidity COPD, Comorbidity Asthma, Comorbidity OSA, Comorbidity CAD, Comorbidity CVA, Comorbidity PAD, Comorbidity HTN, Comorbidity Arrhythmia, Comorbidity Hyperlipid, Comorbidity PE, Comorbidity Heterotaxy, Comorbidity Asplenia, Comorbidity CKD, Comorbidity CA, PVR, Sum of Comorbidities, Any Comorbidity.

Ensemble feature selection[19] based on recursive feature elimination (RFE)[20,21] and a linear SVM[22] as the estimator, was used to ensure robust identification of the smallest set of clinical features (signature), from the clinical features, that best describe each subgroup. RFE feature ranking was based on absolute weights of features from the SVM, which quantifies the contribution or importance of each feature towards the multivariate construction of a hyperplane separating the subgroups. The regularisation parameter of the SVMs was set to C=1. The discovery dataset used for feature selection was resampled without replacement into 500 subsamples (90% of samples), for each subgroup over signature sizes (s) ranging between s=1 to 20. Each

resampled dataset was further divided into bootstrap samples (*k*), with $k=50$. Feature values of each bootstrap sample were normalised to improve feature selection performance. RFE-SVM[20] was used to rank features for each bootstrap sample, and an aggregate rank was calculated for each feature using all *k* bootstrap rankings. This process was repeated over all resampled datasets, resulting in 500 candidate signatures for each signature size, *s*. Each candidate signature was then used to develop a classification model, which was then trained on the discovery dataset to discriminate between a given subgroup from all other subgroups. Classification models were built using support vector machines (SVM)[23], random forest (RF)[24], logistic regression (LR)[25], and k-nearest neighbour (KNN)[26].

LR was implemented using sklearn.linear_model.LogisticRegression using l2 penalty and default values used for all other parameters. SVM was implemented using sklearn.svm.LinearSVC with regularisation parameter C set to 1, and default values used for all other parameters. RF was implemented using sklearn.ensemble.RandomForestClassifier with default values used for all other parameters. kNN was implemented using sklearn.neighbors.KNeighborsClassifier with a number of neighbours (n_neighbors) set to 5 and default values used for all other parameters.

For feature selection tasks, we used ensemble feature selection based on recursive feature elimination (RFE) technique. RFE is a backward feature elimination technique that iteratively prunes the least informative feature(s) from a training dataset. A RFE based on a linear SVM starts by using all features to train an SVM model and ranks all features according to importance. The least ranked feature is removed from the training dataset and the SVM model refitted. This is iteratively done until only the required number of features remain. All features are also ranked according to importance.

Ensemble feature selection aggregates several feature rankings into a single consensus feature ranking to ensure robustness of the feature selection process and of selected features. Feature importance measures used for feature ranking are based on the hyperplane weight vector of a linear support vector machine (SVM). The weight vector quantifies the contribution of each feature to the construction of the hyperplane, and is used for ranking features according to importance.

Ensemble feature selection56 based on recursive feature elimination (RFE)57,58 and a linear SVM59 as the estimator, was used to ensure robust identification of the smallest set of clinical features (signature) that best describe each subgroup. RFE feature ranking was based on absolute weights of features from the SVM, which quantifies the contribution or importance of each feature towards the multivariate construction of a hyperplane separating the subgroups. The regularisation parameter of the SVMs was set to C=1. The discovery dataset used for feature selection was resampled without replacement into 500 subsamples (90% of samples), for each subgroup over signature sizes (s) ranging between s=1 to 20. Each resampled dataset was further divided into bootstrap samples (k), with k=50. Feature values of each bootstrap sample were normalised to improve feature selection performance. RFE-SVM57 was used to rank features for each bootstrap sample, and an aggregate rank was calculated for each feature using all k bootstrap rankings. The set of feature rankings R, aggregated over all bootstrap samples, is calculated as,

$$R = \left(\sum_{i=1}^{k} r_i^1, \sum_{i=1}^{k} r_i^2 \ldots\ldots \sum_{i=1}^{k} r_i^{N-1}, \sum_{i=1}^{k} r_i^N\right) \quad (1)$$

where k is the number of bootstrap samples, N is the total number of features in the dataset, and r_i^n is the rank of feature n in bootstrap sample i. This process was repeated over all resampled datasets, resulting in 500 candidate signatures for each signature size, s.

The candidate signature that obtained the best performance was selected. This process was repeated for all signature sizes, *s*=1 to $s=20$, for subgroups I, II and v. A final signature for each subgroup was selected based on a compromise between the fewest number of features ($s=1$ *to* $s=20$) and classification performance. Final selected signatures for each of the subgroups were pooled to create a composite signature, which was then used to develop a multi-class classification model. The model was trained on the discovery dataset to discriminate between subgroups I, II and V, used to predict subgroup membership of an unseen validation dataset. The predicted subgroup membership was then used to calculate survival of predicted subgroups. Survival of the

predicted subgroups was compared to known survival of subgroups in the discovery dataset for validation purposes.

Differential expression analysis

Differential expression analysis was performed between patients' subgroups. The raw un-normalised counts which were the output of the Salmon quantification were used. The DESeqDataSetFromTximport function was used to create an input data-set for DESeq2 which included the raw count data, and the subgroup membership for each sample. Rows with fewer than a total of 10 counts were excluded in order to decrease computing time.

Utilizing the apeglm R package the log fold change (LFC) shrinkage was performed on the results in order to reduce noise from genes with low counts, while retaining genes with large fold changes. This is an alternative to introducing filtering thresholds or pseudocounts which have disadvantages such as resulting in the loss of genes with true expression differences. This method was used to create pairwise comparisons of gene LFC between the control subgroup and each patient subgroup. Using the LFC data, genes with a log2fold change of greater than +/-1.5 were selected, and ranked by their p-value. The LFC data used ensembl92 gene IDs, so the biomaRt R package was used to add HUGO Gene Nomenclature Committee (HGNC) gene names and a brief description to allow for easier identification of genes of interest.

Secondary clustering analysis with healthy volunteers

All RNA-seq samples (508) are utilised along with the genes that provide the most information when attempting to predict whether each sample belongs to a patient or a healthy volunteer. We used the patient and control labels as ground truth. Utilizing the labels, we ranked the genes according to the amount of information they contribute in distinguishing the two classes. To determine the amount of information each gene contributed towards separating IPAH and healthy samples we used the Information Gain Criterion from the Biocomb R package. The 25,955 genes were scored and ranked. To generate the candidate gene sets for the determination of the most stable one, multiple subsets of the top ranked genes were extracted, each time increasing the size by 50. While investigating the differences between disease and healthy samples we can utilise the only ground truth we have established, the partitioning of the samples into patient and control groups. This knowledge enables the selection of the number of subgroups (k) to be based on the average subgroup purity of each k and compare them to select the k with the highest average purity.

Additional clustering pipeline of IPAH patients

To estimate the impact the 33 HPAH patients had on our main clustering pipeline, we examined their distribution across subgroups and ran an additional clustering pipeline including exclusively the 313 IPAH samples. As in the main pipeline, we utilised spectral clustering with the rbfdot kernel, the 300 most variable genes and identical preprocessing (section **Sample and gene selection preprocessing**).

Supplementary Tables

Supplementary Table 1 | Distribution of HPAH samples across the 5 subgroups and their proportion in each subgroup.

I	II	III	IV	V
12(9.3%)	6(5.35%)	3(15.7%)	1(10%)	11(12.3%)

Supplementary Table 2 | Bonferroni adjusted p-values for various white blood cell counts.

Subgroup Pairs	*Lymphocytes*	*Eosinophils*	*Monocytes*	*Neutrophils*	*Neutrophils / Lymphocytes*
**I - II**	0.13	0.18	0.0076	7.2e-12	0.0061
**I - III**	0.14	1	1	8.0e-04	0.1100
**I - IV**	1	1	0.0390	8.7e-03	1
**I - V**	1	1	0.5900	1.3e-03	1
**II - III**	0.47	1	1	1	1
**II - IV**	1	0.87	1	3.2e-10	1
**II - V**	1	1	1	4.4e-04	0.23
**III - IV**	1	1	1	2.7e-06	1
**III - V**	0.16	1	1	1.3e-01	0.0670
**IV - V**	1	1	1	6.4e-08	1

Supplementary Table 3 | The distribution of BMPR2 mutations across all patient subgroups (n=357).

	**I**	**II**	**III**	**IV**	**V**
BMPR2	22 (20.5%)	23 (26.1%)	4 (26.6%)	4 (50%)	18 (26.4%)
Not BMPR2	107	88	15	8	68

Supplementary Table 4 | All fold changes p values of the gene signature for subgroups calculated using a two-sided t-test.

Gene	P value	Gene	P value
ALAS2	1.60E-12	MIR5195	2.36E-13
LTF	5.40E-06	IGKJ4.43	1.92E-17
CRISP3	4.99E-07	IGHM	8.50E-16
CTSG	1.53E-05	IGHV4.39	2.69E-12
RP11.20D14.6	1.73E-08	IGLV2.14	6.82E-22
RP11.678G14.3	4.53E-09	IGKV1.27	2.42E-10
NPRL3	0.006121994	IGHV3.48	2.55E-17
CH17.296N19.1	6.31E-05	IGLV6.57	2.01E-11
RP1.229K20.9	0.002453699	IGLV7.43	5.59E-18
AC131056.3	0.08654707	IGHV2.5	4.21E-08
MT.RNR1	0.4689423	MIR5195	2.36E-13
NOG	1.05E-11	IGKJ4.43	1.92E-17

Supplementary Table 5 | Correlations between discovery, validation and external validation cohorts. In the discovery set, Spearman correlations were calculated between RNA-req TPM values, the validation set between negative delta Cts values with GAPDH used as the endogenous control gene. Green cells denote agreement in the directionality of gene-clinical variable correlations between the validation sets and the discovery set. Red cells denote disagreement/opposite correlation between the two data sets. Notation of (**) denotes a p-value of less than 0.01, (***) denotes a p-value of less than 0.001 (using asymptotic approximated p-value by using the t distribution , while no stars denote non-significant p-values.

Genes	***Discovery [n= 359]***			***Validation [n =91]***			***External Validation [n =32]***		
	Age	***BMI***	***SixMWD***	***Age***	***BMI***	***SixMWD***	***Age***	***BMI***	***SixMWD***
**ALAS2**	0.2***	0.38***	-0.32***	-0.006	0.06	-0.1	-0.08	0.31	-0.35
**C4BPA**	0.01	0.02	-0.07	0.14	-0.11	-0.13	0.02	-0.19	0.25
**CRISP3**	0.16*	0.14	-0.16*	0.11	0.016	-0.22	-0.07	0.14	0.05
**CTSG**	0.19***	0.14*	-0.12*	0.07	0.06	-0.17	0.06	0.19	-0.12
**IFI27**	0.19**	0.16*	-0.22***	0.24*	0.004	-0.28*	0.1	0.1	-0.32
**IGHM**	-0.29***	-0.18***	0.2***	-0.42***	-0.21*	0.18	-0.15	0.33	-0.02
**IGHV3.48**	-0.27***	-0.08	0.14*	-0.31	0.34	-0.5*	-0.11	0.22	0.12
**IGKV2.24**	-0.25***	-0.26***	0.24***	-0.45***	-0.17	0.11	-0.07	0.14	0.15
**IGLV6.57**	-0.21***	-0.16*	0.15*	-0.5***	-0.08	0.16	-0.31	0.32	0.04
**LTF**	0.18**	0.19***	-0.13*	0.08	0.12	-0.23*	0.01	0.08	0.01
**NEBL**	0.01	0.05	0.01	-0.09	-0.01	-0.23	0.03	0.12	-0.16
**NOG**	-0.44***	-0.19***	0.2***	-0.58***	-0.2*	0.18	-0.13	0.02	0.14
**NPRL3**	0.05	0.18*	-0.13*	-0.002	-0.09	-0.1	0.03	-0.06	-0.41
**PI3**	0.15**	0.25***	-0.17**	0.1	0.04	-0.21	-0.13	-0.02	0.31
**SMIM11A**	-0.03	-0.2***	0.12*	-0.24*	-0.13	0.06	0.1	-0.04	0.18

Supplementary Table 6 | The clustering algorithms, their approach category and the various distance measures tested.

**Clustering algorithms**	**Category**	**Distance measures**
K-means	Partitioning	rbfdot, polydot, tanhdot, laplacedot
Hierarchical	Hierarchical	euclidean, manhattan, minkowski, canberra
Spectral	Graph Theory	rbfdot, polydot, tanhdot, laplacedot

Supplementary Figures

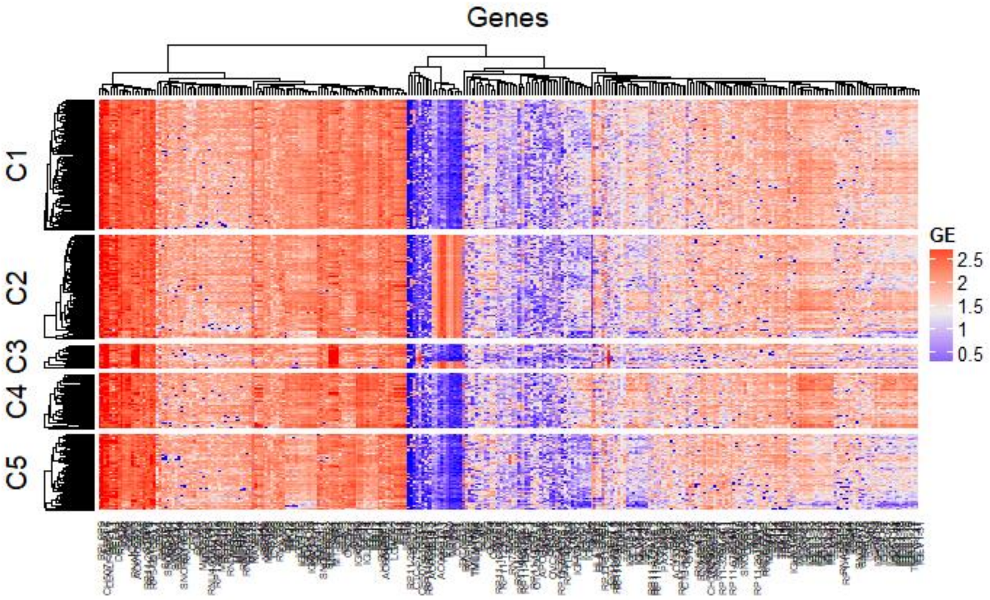

Supplementary Figure 1: Heatmap of gene expression after clustering with 11 male genes included. Separation of subgroups consisted solely of males (C2 and C3) or females (C1, C4, C5), thus obstructing the capturing of the disease signal. 11 male specific genes (PRKY, TTTY15, AC006032.1, RPS4Y1, EIF1AY, KDM5D, TXLNG2P, USP9Y, ZFY, DDX3Y, UTY) were observed to drive the initial clustering of genomic expression profiles.

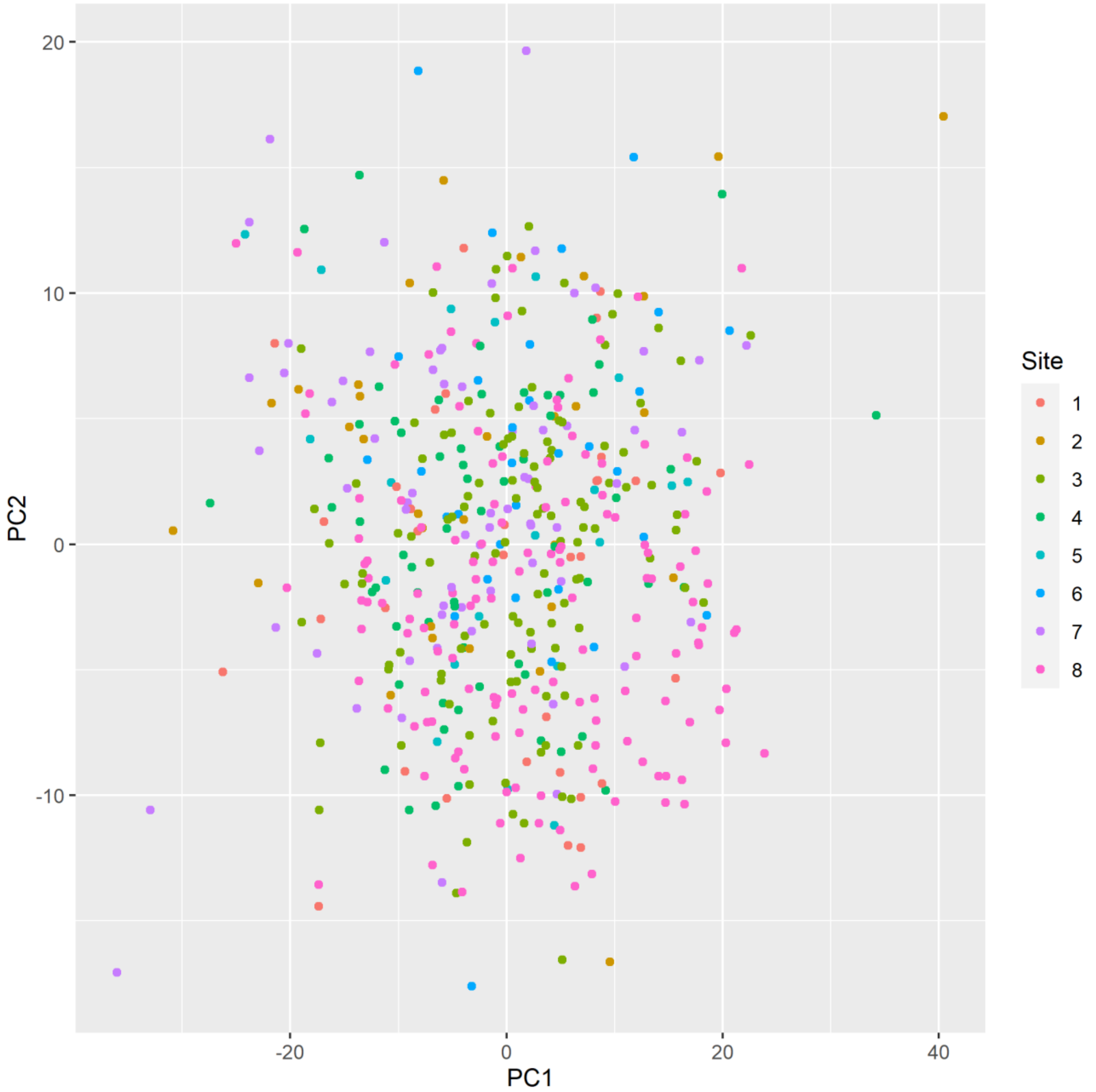

Supplementary Figure 2: Plot of the first two principal components of the RNA-seq data derived from the 10,000 most variable genes according to IQR in our dataset. Each dot represents a distinct sample in the dataset coloured according to the institute that provided that sample. No discernible effect is seen due to the sample collection site.

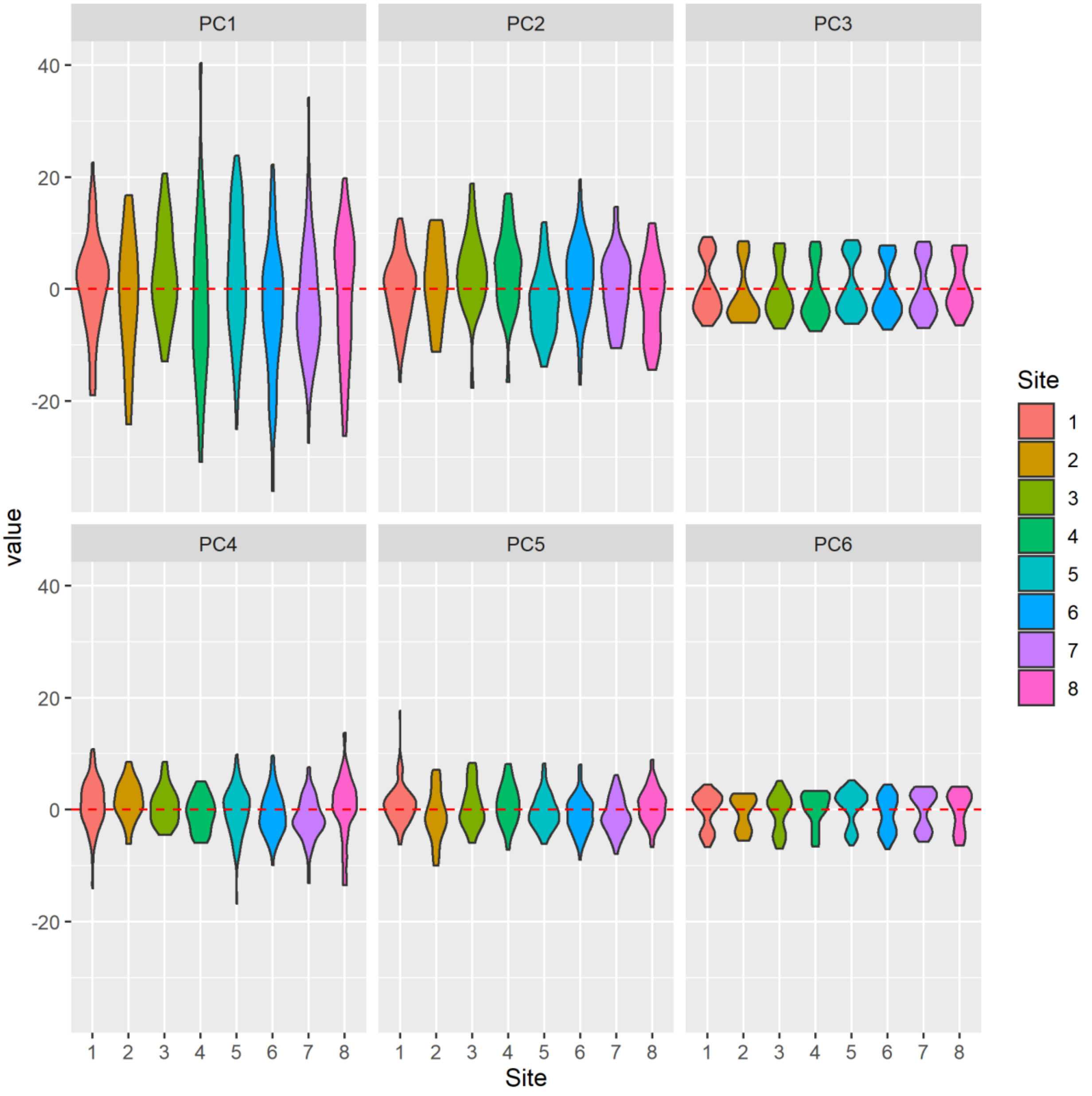

Supplementary Figure 3: Boxplots showing the distribution of the first eight principal components of the RNA-seq dataset (n = 10,000 most variable genes) grouped according to the Site that provided the sample. No discernible effect is seen due to sample collection site. Red line represents the median, top and bottom bounds of the box represent min and max values.

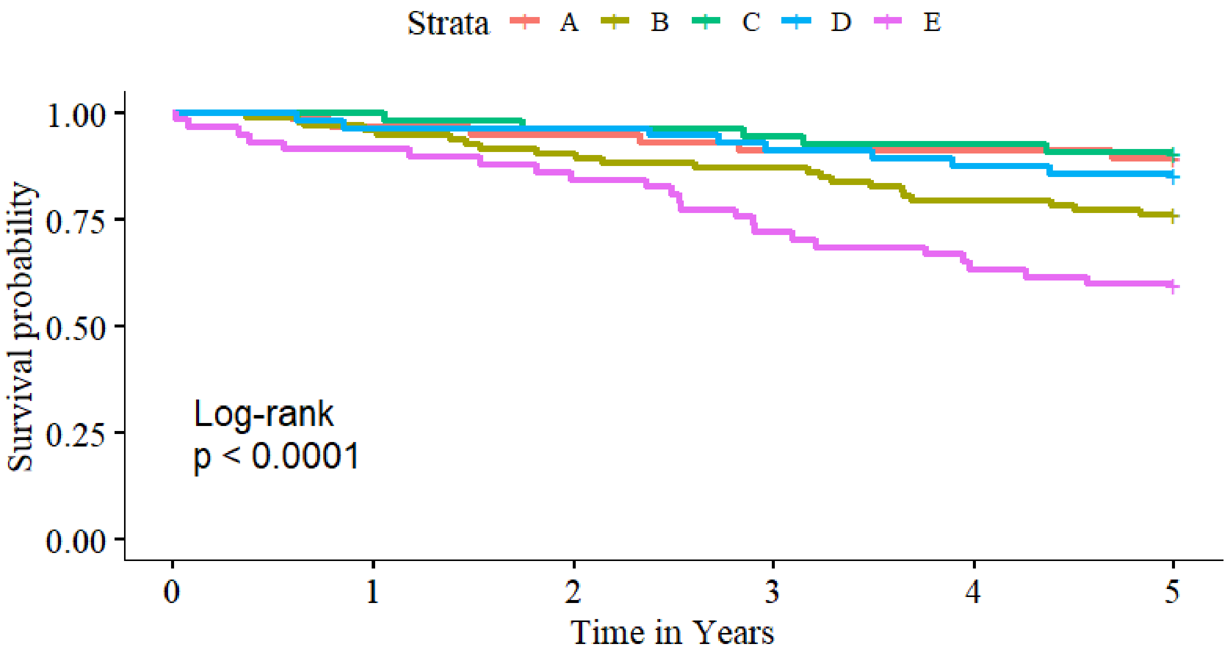

Supplementary Figure 4: Survival of patients in clusters(A, B, C, D, E) created by clustering only IPAH samples with two-sided log rank test p-value.

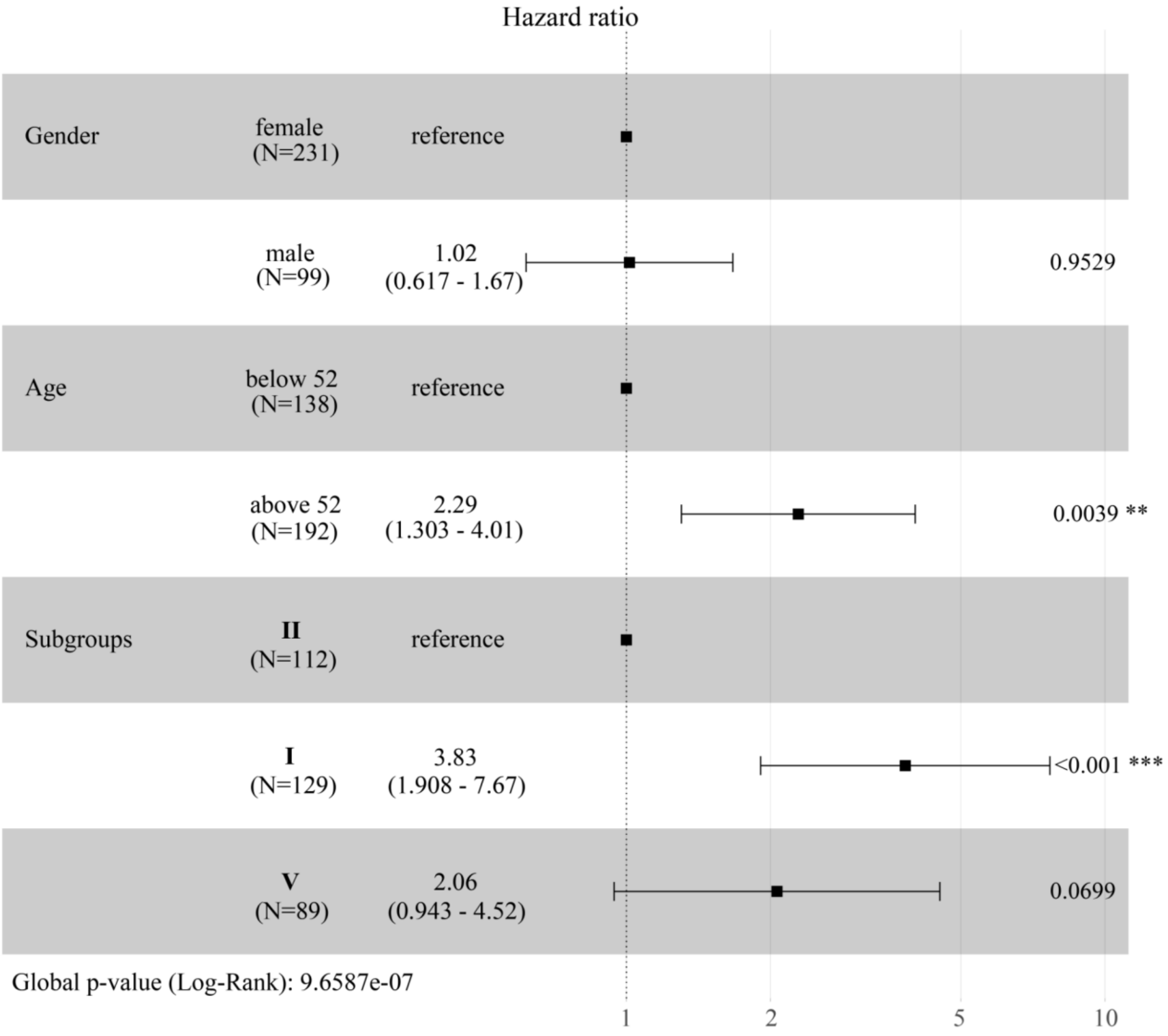

Supplementary Figure 5: Hazard Ratio of discovery cohort clustering adjusted for gender and age category of patients. Notation of (**) denotes a two-sided log rank test p-value of less than 0.01, (***) denotes a p-value of less than 0.001, while no stars denote non-significant p-values. Data are presented as median values and error bars as 95% confidence intervals. Gender did not reveal any relationship with survival while an age over 52 was significantly associated with poor survival (HR=2.29). The most significant association with poor survival was found for patients classified in subgroup I.

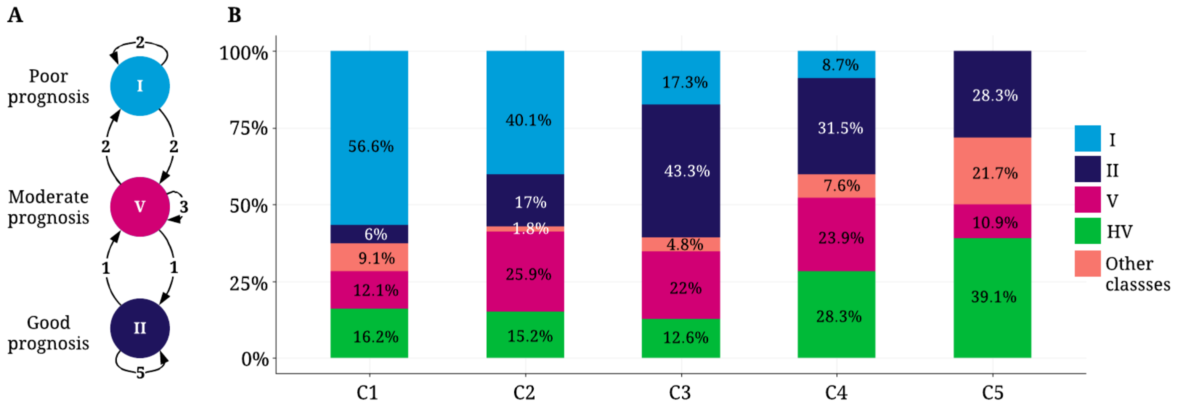

Supplementary Figure 6 | Step-wise progression of IPAH. From healthy individuals to subgroup II, then V, then I. (A) longitudinal samples change across subgroups with different prognoses. (B) The five subgroups (C1-C5) from our secondary clustering pipeline which included patients and healthy volunteers. Each subgroup contains subgroups from our original clustering (I, II, V and the other classes -III and IV-) and healthy volunteers (HV).

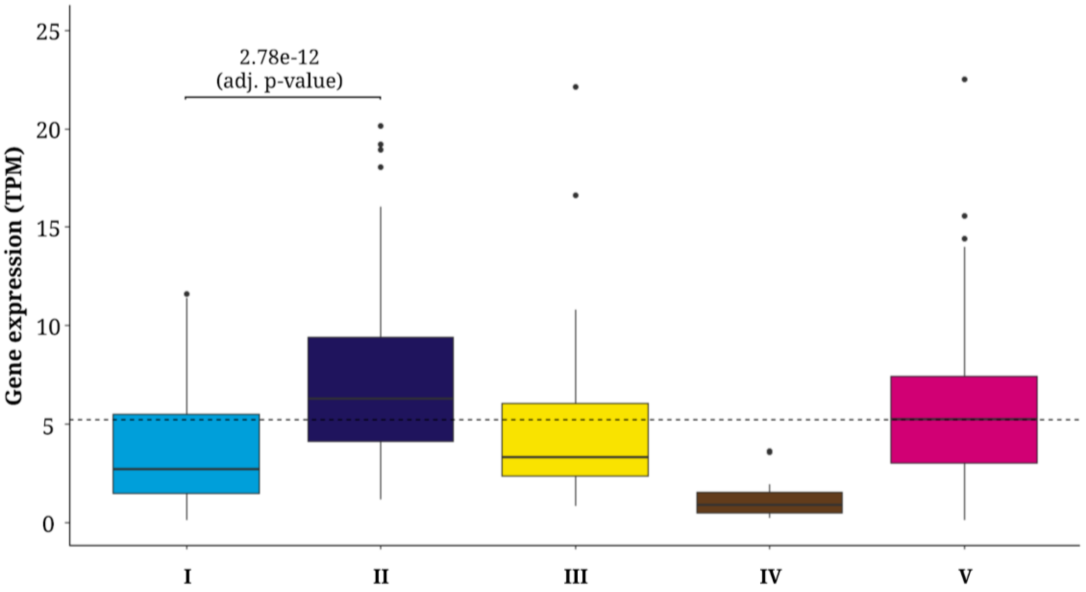

Supplementary Figure 7: Noggin presents significantly higher expression in the high survival/low risk subgroup II. Furthermore, it is shown to be upregulated in the same subgroup based on our LASSO regression analysis. Boxplots were calculated for I (n=134), II (n=119), III (n=19), IV (n=10) and V(n=98). Vertical center line represents the median, top and bottom bounds of the box represent the first and third quartile, while the tips of the whiskers represent min and max values.

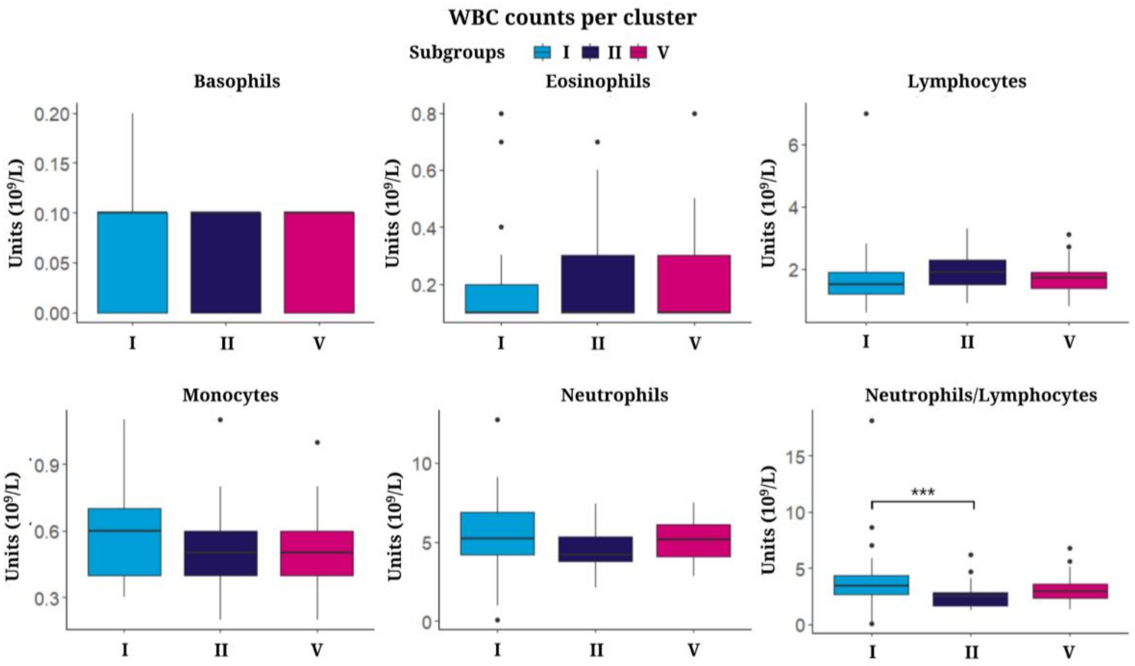

Supplementary Figure 8: Quantity of various types of white blood cells across subgroups I (n=41), II (n=43) and V(n=28). Vertical center line represents the median, top and bottom bounds of the box represent the first and third quartile, while the tips of the whiskers represent min and max values.

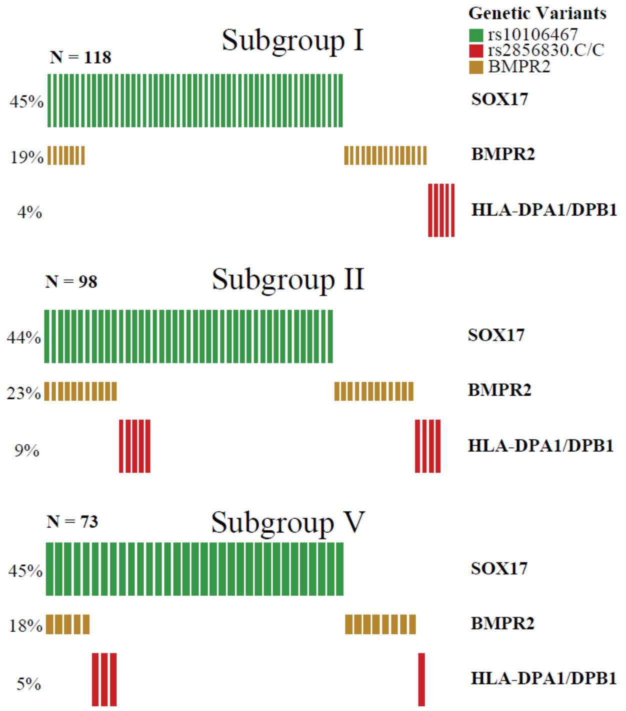

Supplementary Figure 9: Oncoprint of variants previously associated with PAH across RNA subgroups. Presence of any pathogenic BMPR2 variant is labeled for each patient while the presence of specific SNPs for SOX17 and HLA-DPA1/DPB1 are labeled.

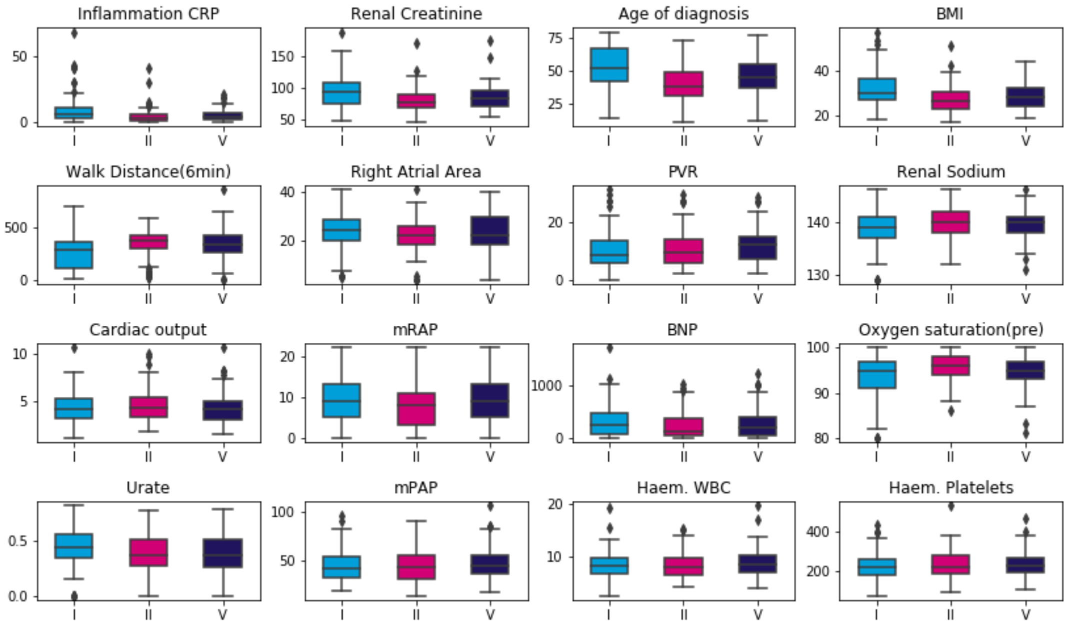

Supplementary Figure 10: Comparative measurements of all clinical variables from feature selection across RNA-based subgroups for subgroups I(n=129), II(n=112) and V(n=89). Vertical center line represents the median, top and bottom bounds of the box represent the first and third quartile, while the tips of the whiskers represent min and max values.

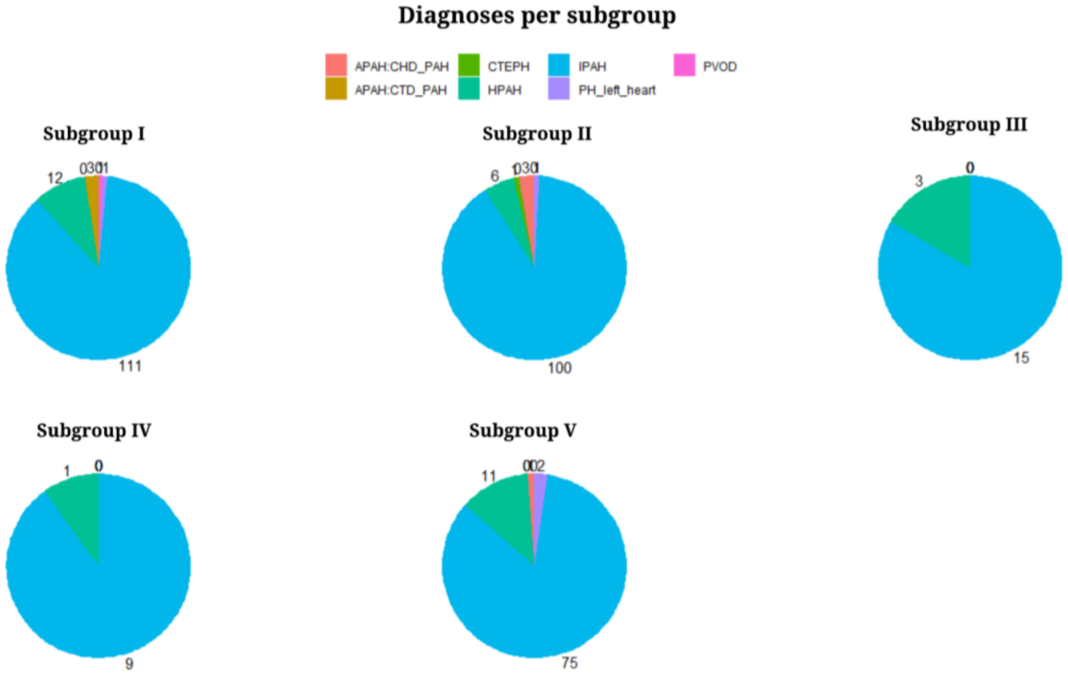

Supplementary Figure 11: Revised diagnoses for each subgroup

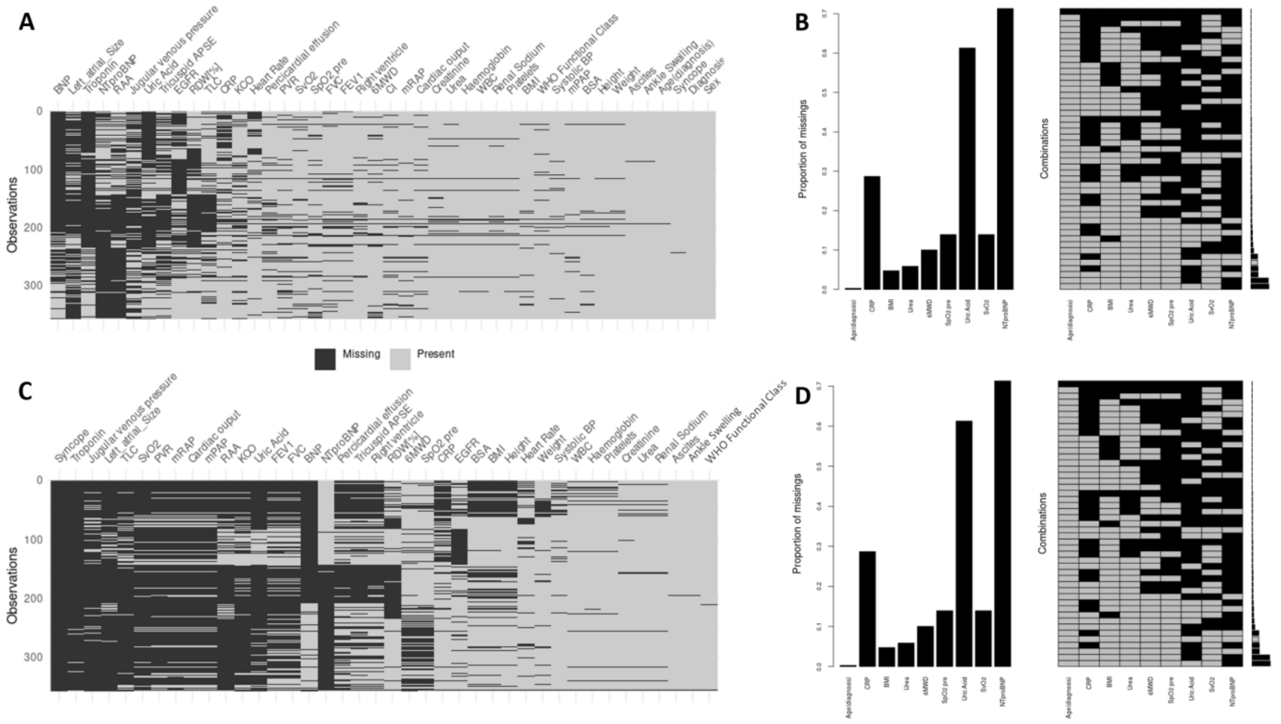

Supplementary Figure 12: A) Heatmap showing missingness across important clinical variables for the diagnostic dataset. B) Barchart showing the proportion of missing data and chart showing the combinations of missing data for the classifier variables from the diagnostic dataset. C) Heatmap showing missingness across important clinical variables for the cohort visit 1 dataset. D) Barchart showing the proportion of missing data and chart showing the combinations of missing data for the classifier variables from the cohort visit 1 dataset.

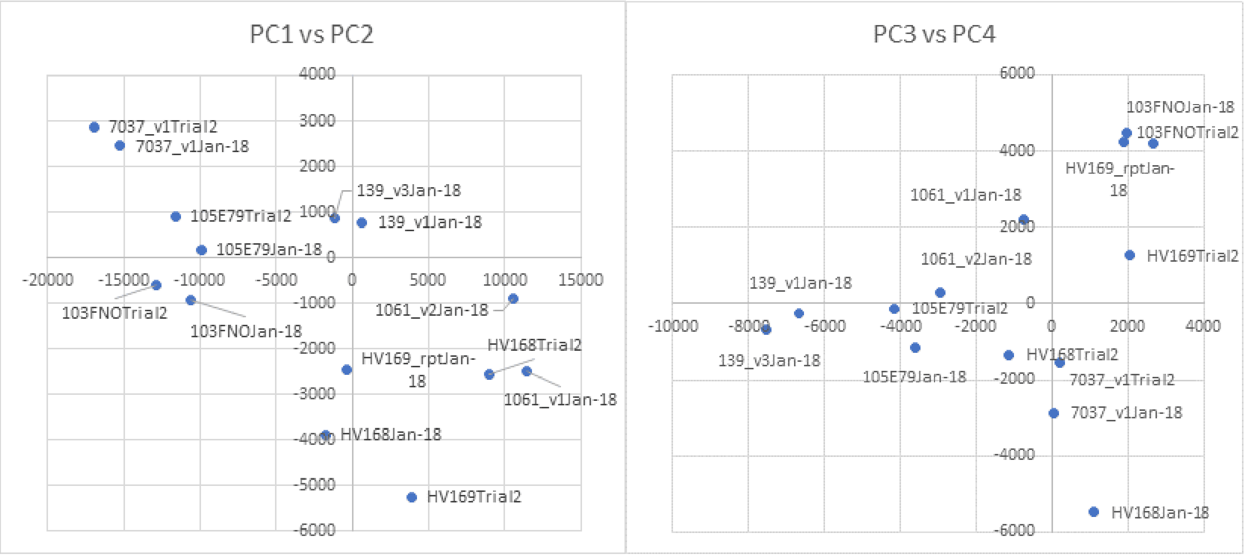

Supplementary Figure 13: Principal components analysis of expression profiles from samples with a second replicate that was RNA sequenced (labeled as "...Trial2"). Both replicates are clustered together according to the first four principal components.

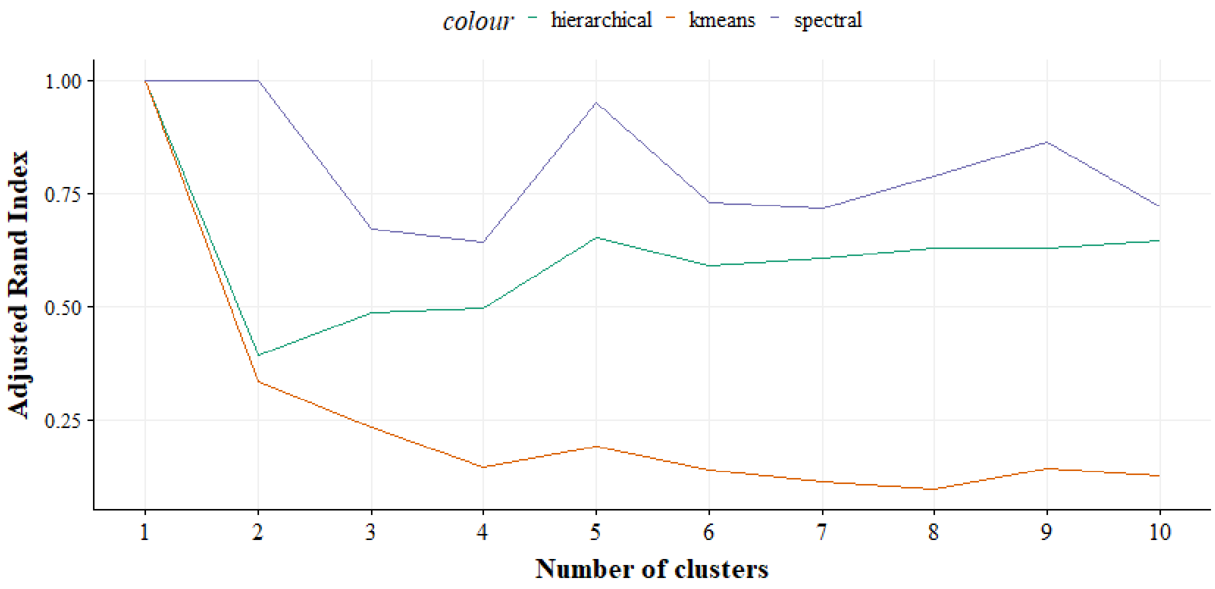

Supplementary Figure 14: The average adjusted rand index (ARI) of three clustering methods: spectral (blue line), hierarchical (green line) and k-means (red line) clustering. For each method 3 different distance measures/kernels were used and their ARI was averaged for each method.

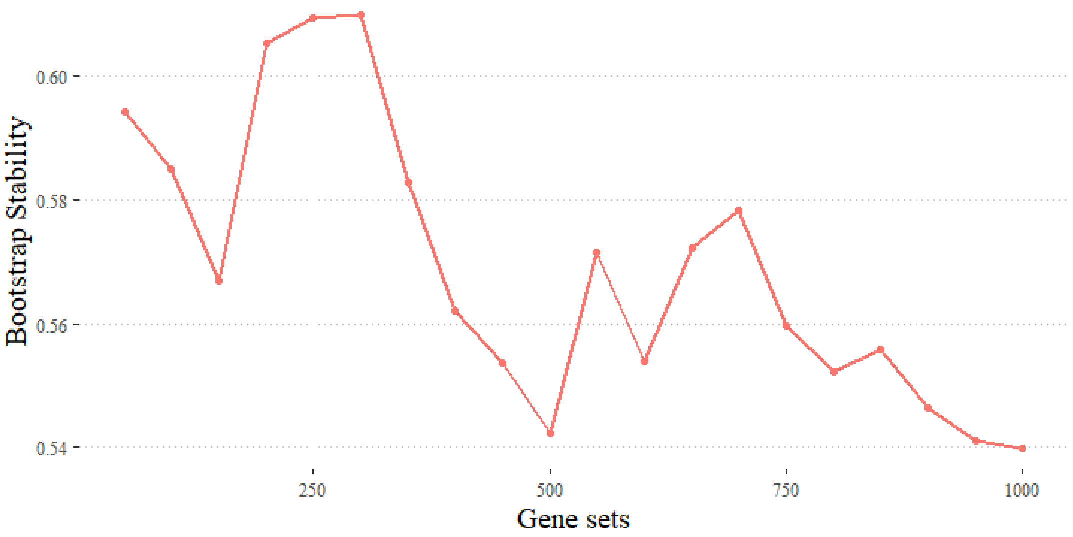

Supplementary Figure 15: The bootstrap resampling describes stability of clustering as a function of increasing size (by 50) of gene sets. The highest stability is observed for the gene set that contains the top 300 variable genes across patients

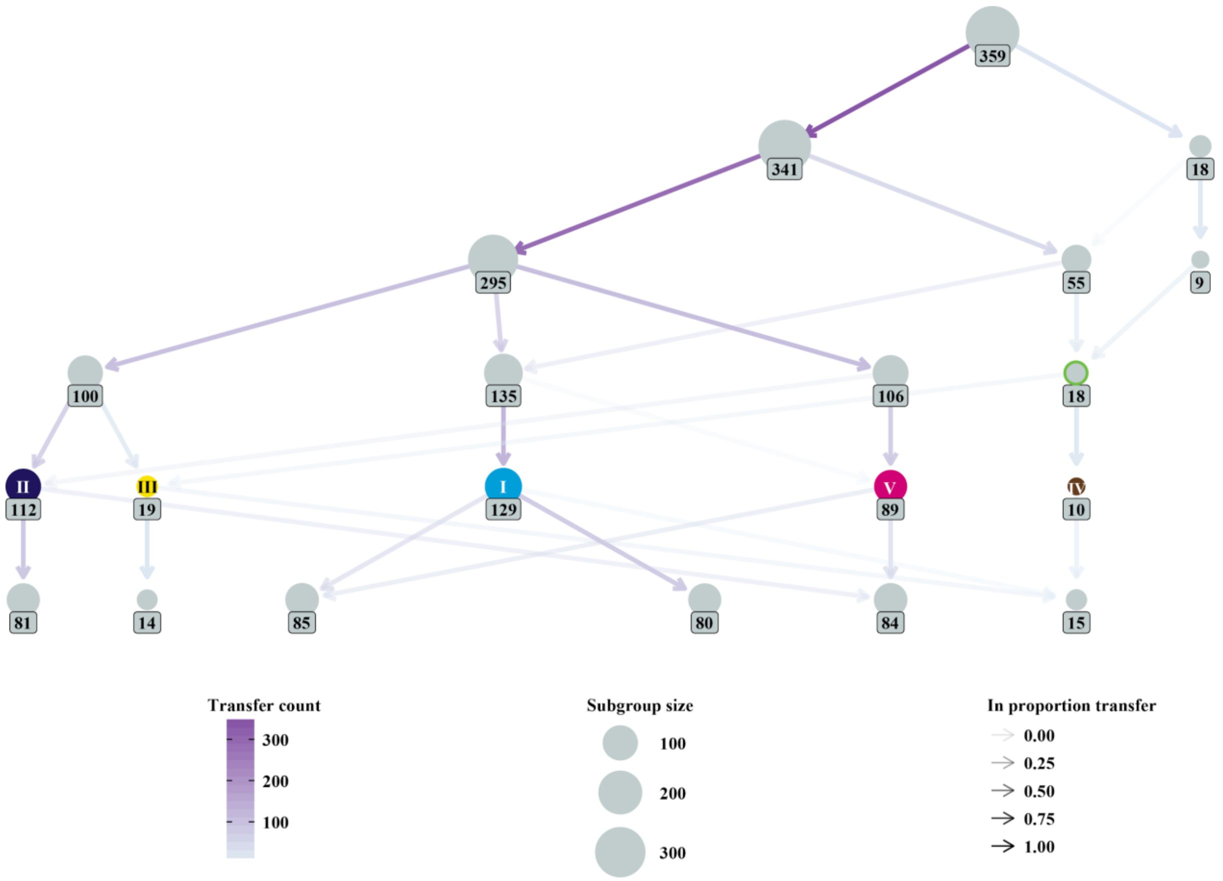

Supplementary Figure 16: Clustree visualisation of the 5 subgroups (colored) discovered by our spectral clustering methodology. Edges represent the transfer of patients between clusterings of different k. Their opaqueness indicates the amount of patients that transferred.

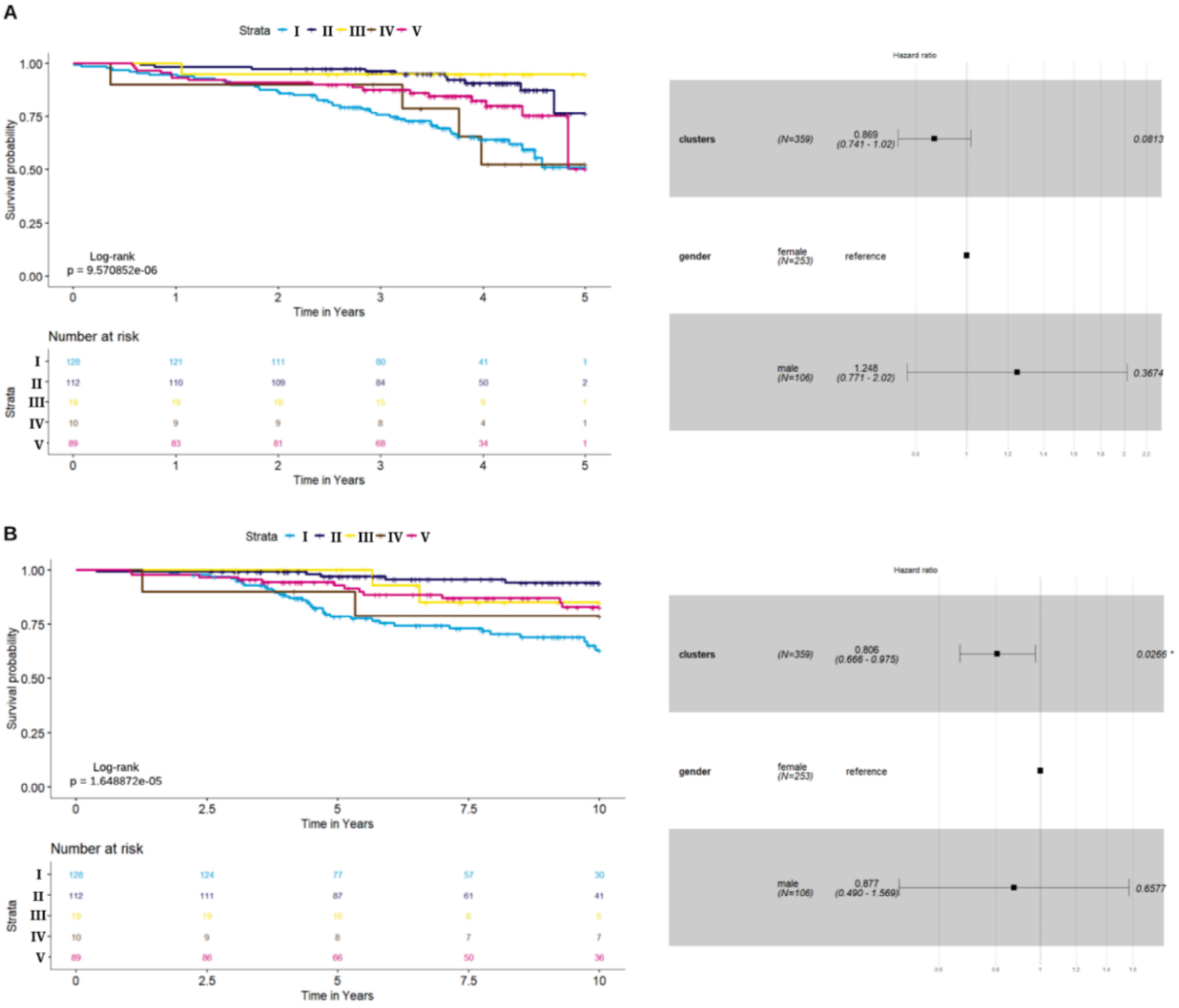

Supplementary Figure 17: Survival curves and gender hazard ratios from Cox Proportional Hazards model using data (A) from sampling and (B) time of diagnosis. Forest plot data are presented as median values and error bars as 95% confidence intervals.

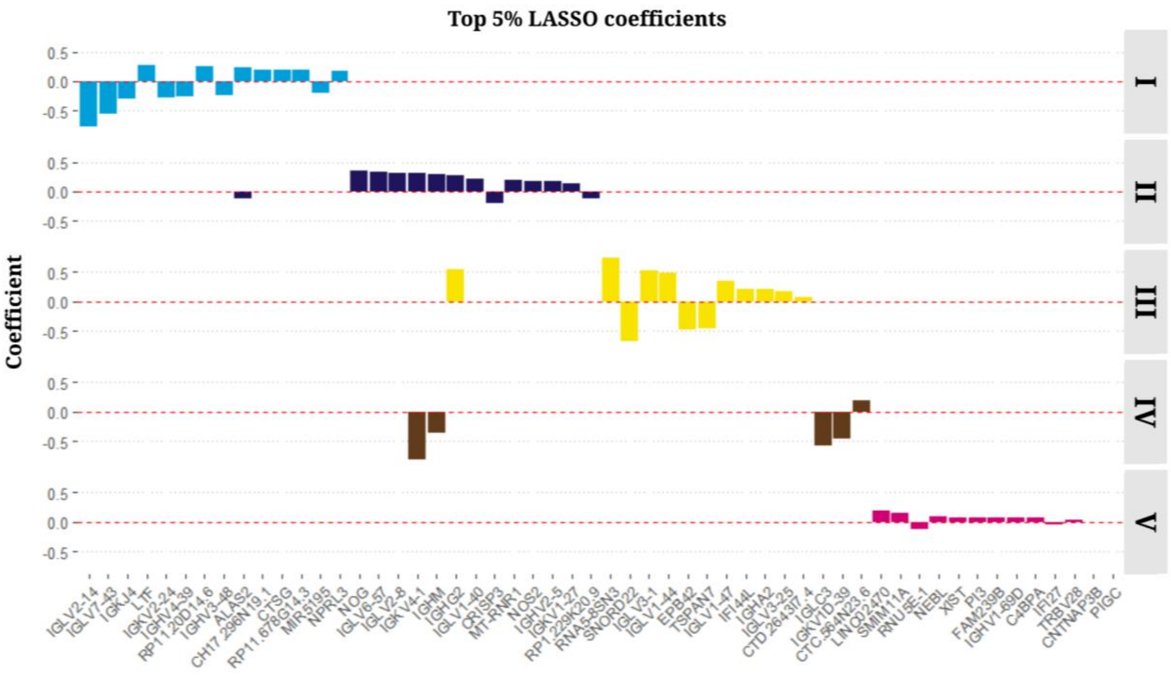

Supplementary Figure 18: Genes with the top 5% of Lasso coefficients showing gene signatures for each subgroup.

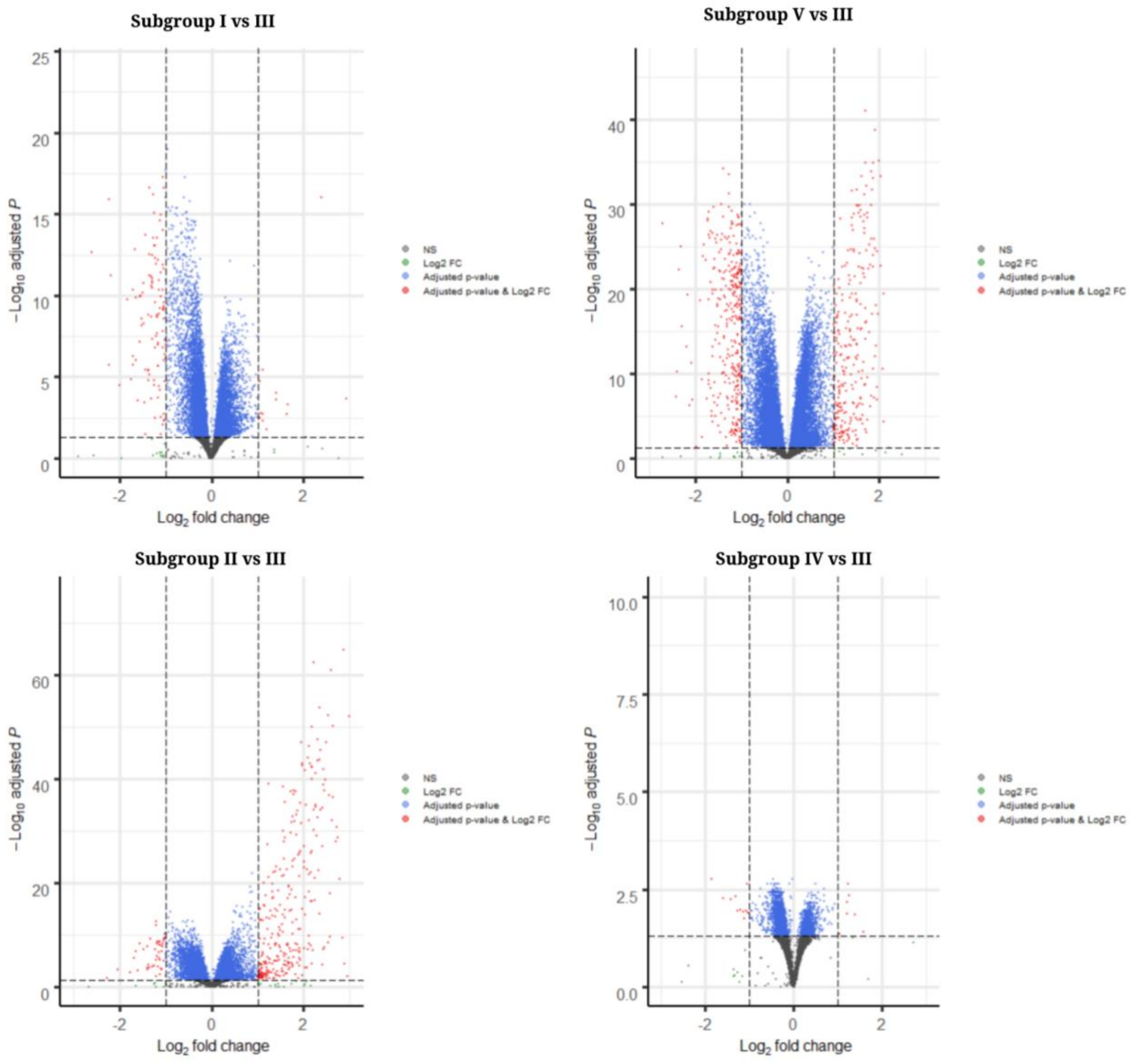

Supplementary Figure 19: Volcano plots of differential expression between pairs of subgroups. Dots coloured in red are genes with > 2 fold change and bonferroni log adjusted two-sided t-test p<0.01.

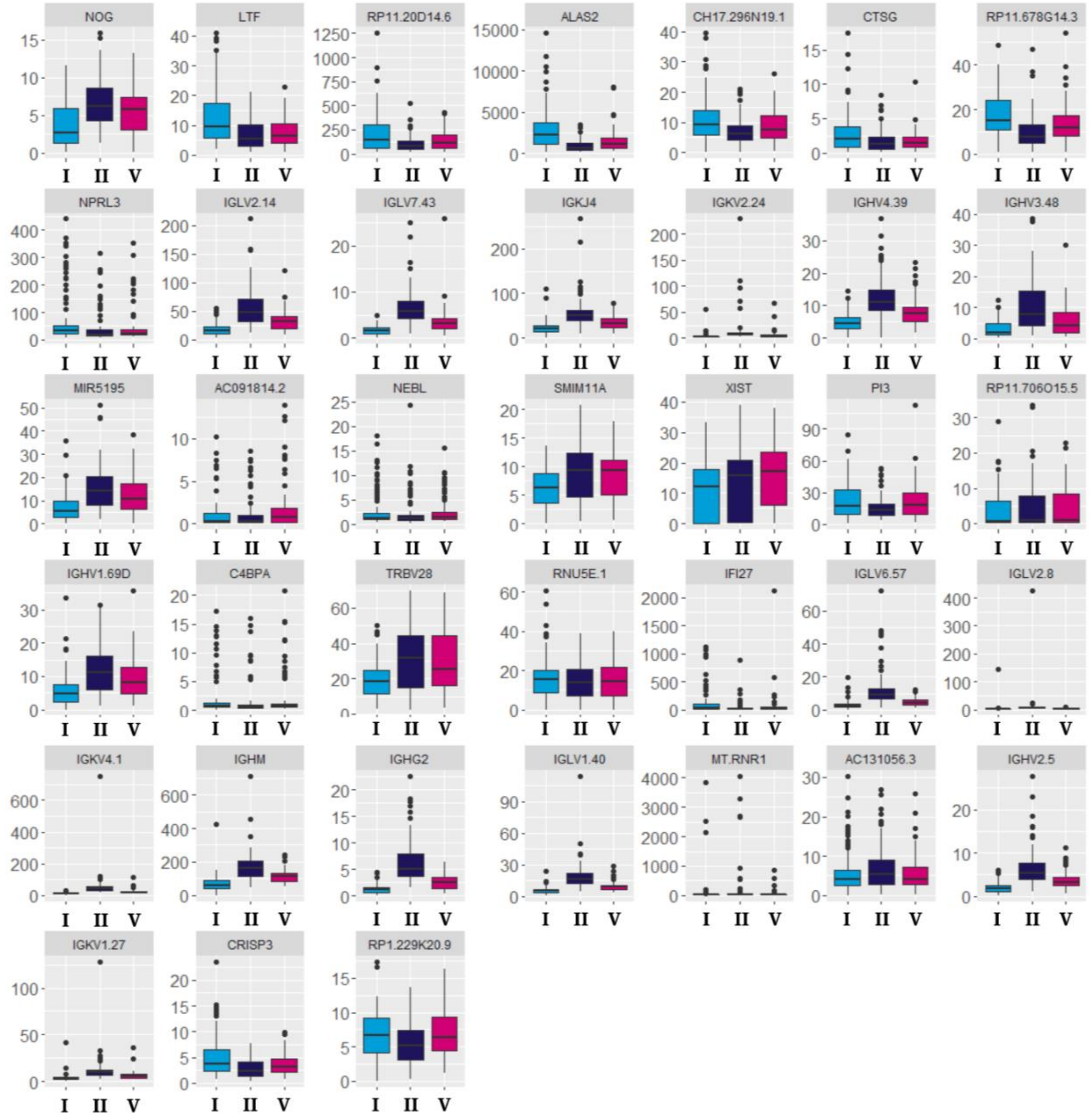

Supplementary Figure 20: Gene signature expression (TPM) across subgroups I(n=129), II(n=112) and V(n=89). Vertical center line represents the median, top and bottom bounds of the box represent the first and third quartile, while the tips of the whiskers represent min and max values.

Supplementary references

1. Rhodes, C. J. *et al.* Whole Blood RNA Profiles Associated with Pulmonary Arterial Hypertension and Clinical Outcome. *Am. J. Respir. Crit. Care Med.* (2020) doi:10.1164/rccm.202003-0510OC.
2. Polikar, R. Ensemble based systems in decision making. *IEEE Circuits and Systems Magazine* vol. 6 21–45 (2006).
3. Sweatt, A. J. *et al.* Discovery of Distinct Immune Phenotypes Using Machine Learning in Pulmonary Arterial Hypertension. *Circ. Res.* **124**, 904–919 (2019).
4. Kherbeck, N. *et al.* The role of inflammation and autoimmunity in the pathophysiology of pulmonary arterial hypertension. *Clin. Rev. Allergy Immunol.* **44**, 31–38 (2013).
5. Hemnes, A. R. & Humbert, M. Pathobiology of pulmonary arterial hypertension: understanding the roads less travelled. *Eur. Respir. Rev.* **26**, (2017).
6. Calinski, T. & Harabasz, J. A dendrite method for cluster analysis. *Communications in Statistics - Theory and Methods* vol. 3 1–27 (1974).
7. Dunn†, J. C. Well-Separated Clusters and Optimal Fuzzy Partitions. *Journal of Cybernetics* vol. 4 95–104 (1974).
8. Pakhira, M. K., Bandyopadhyay, S. & Maulik, U. Validity index for crisp and fuzzy clusters. *Pattern Recognition* vol. 37 487–501 (2004).
9. Kendall, M. G. A New Measure of Rank Correlation. *Biometrika* vol. 30 81 (1938).
10. Baker, F. B. & Hubert, L. J. Measuring the Power of Hierarchical Cluster Analysis. *Journal of the American Statistical Association* vol. 70 31–38 (1975).
11. Hubert, L. J. & Levin, J. R. A general statistical framework for assessing categorical clustering in free recall. *Psychological Bulletin* vol. 83 1072–1080 (1976).
12. Davies, D. L. & Bouldin, D. W. A Cluster Separation Measure. *IEEE Transactions on Pattern Analysis and Machine Intelligence* vol. PAMI-1 224–227 (1979).
13. Halkidi, M., Batistakis, Y. & Vazirgiannis, M. Clustering algorithms and validity measures. *Proceedings Thirteenth International Conference on Scientific and Statistical Database Management. SSDBM 2001* doi:10.1109/ssdm.2001.938534.
14. Rohlf, F. J. Methods of Comparing Classifications. *Annual Review of Ecology and Systematics* vol. 5 101–113 (1974).

15. Rousseeuw, P. J. Silhouettes: A graphical aid to the interpretation and validation of cluster analysis. *Journal of Computational and Applied Mathematics* vol. 20 53–65 (1987).

16. Halkidi, M., Batistakis, Y. & Vazirgiannis, M. On Clustering Validation Techniques. *J. Intell. Inf. Syst.* **17**, 107–145 (2001).

17. Song, Y. Class compactness for data clustering. in *2010 IEEE International Conference on Information Reuse & Integration* 86–91 (IEEE, 2010).

18. Goel, M. K., Khanna, P. & Kishore, J. Understanding survival analysis: Kaplan-Meier estimate. *Int. J. Ayurveda Res.* **1**, 274–278 (2010).

19. Abeel, T., Helleputte, T., Van de Peer, Y., Dupont, P. & Saeys, Y. Robust biomarker identification for cancer diagnosis with ensemble feature selection methods. *Bioinformatics* vol. 26 392–398 (2010).

20. Duan, K.-B., Rajapakse, J. C., Wang, H. & Azuaje, F. Multiple SVM-RFE for Gene Selection in Cancer Classification With Expression Data. *IEEE Transactions on Nanobioscience* vol. 4 228–234 (2005).

21. Granitto, P. M., Furlanello, C., Biasioli, F. & Gasperi, F. Recursive feature elimination with random forest for PTR-MS analysis of agroindustrial products. *Chemometrics Intellig. Lab. Syst.* **83**, 83–90 (2006).

22. Williams, J. A., Weakley, A., Cook, D. J. & Schmitter-Edgecombe, M. Machine learning techniques for diagnostic differentiation of mild cognitive impairment and dementia. in *Workshops at the twenty-seventh AAAI conference on artificial intelligence* (2013).

23. Choi, E., Schuetz, A., Stewart, W. F. & Sun, J. Using recurrent neural network models for early detection of heart failure onset. *J. Am. Med. Inform. Assoc.* **24**, 361–370 (2017).

24. Breiman, L. Machine Learning. vol. 45 5–32 (2001).

25. Hosmer, D. W., Jr., Lemeshow, S. & Cook, E. D. *Applied Logistic Regression, Second Edition: Book and Solutions Manual Set*. (Wiley-Interscience, 2001).

26. Peterson, L. K-nearest neighbor. *Scholarpedia J.* **4**, 1883 (2009).
