# Supplementary material for: Biological heterogeneity in idiopathic pulmonary arterial hypertension identified through unsupervised transcriptomic profiling of whole blood
Source: Nat Commun. 2021 Dec 7;12:7104. doi: 10.1038/s41467-021-27326-0 (PMC8651638; doi:10.1038/s41467-021-27326-0)
Supplement: Supplementary file 3 — Description of Additional Supplementary Files [file 41467_2021_27326_MOESM3_ESM.docx]

File Name: Supplementary Data 1

Description: Values of 14 internal indexes used to estimate the optimal number of subclasses (k)

File Name: Supplementary Data 2

Description: Full demographics table for discovery cohort

File Name: Supplementary Data 3

Description: All LASSO generated coefficients per subgroup
