# Supplementary material for: Biological heterogeneity in idiopathic pulmonary arterial hypertension identified through unsupervised transcriptomic profiling of whole blood
Source: Nat Commun. 2021 Dec 7;12:7104. doi: 10.1038/s41467-021-27326-0 (PMC8651638; doi:10.1038/s41467-021-27326-0)
Supplement: Supplementary file 7 — Reporting Summary [file 41467_2021_27326_MOESM7_ESM.pdf]

nature research

Corresponding author(s): Allan Lawrie and Dennis Wang

Last updated by author(s): Oct 6th 2021

Reporting Summary

Nature Research wishes to improve the reproducibility of the work that we publish. This form provides structure for consistency and transparency in reporting. For further information on Nature Research policies, see our Editorial Policies and the Editorial Policy Checklist.

Statistics

For all statistical analyses, confirm that the following items are present in the figure legend, table legend, main text, or Methods section.

n/a	Confirmed	
☐	☒	The exact sample size (n) for each experimental group/condition, given as a discrete number and unit of measurement
☐	☒	A statement on whether measurements were taken from distinct samples or whether the same sample was measured repeatedly
☐	☒	The statistical test(s) used AND whether they are one- or two-sided<br>*Only common tests should be described solely by name; describe more complex techniques in the Methods section.*
☐	☒	A description of all covariates tested
☐	☒	A description of any assumptions or corrections, such as tests of normality and adjustment for multiple comparisons
☐	☒	A full description of the statistical parameters including central tendency (e.g. means) or other basic estimates (e.g. regression coefficient) AND variation (e.g. standard deviation) or associated estimates of uncertainty (e.g. confidence intervals)
☐	☒	For null hypothesis testing, the test statistic (e.g. F, t, r) with confidence intervals, effect sizes, degrees of freedom and P value noted<br>*Give P values as exact values whenever suitable.*
☒	☐	For Bayesian analysis, information on the choice of priors and Markov chain Monte Carlo settings
☐	☒	For hierarchical and complex designs, identification of the appropriate level for tests and full reporting of outcomes
☐	☒	Estimates of effect sizes (e.g. Cohen's d, Pearson's r), indicating how they were calculated

Our web collection on statistics for biologists contains articles on many of the points above.

Software and code

Policy information about availability of computer code

Data collection	Pseudonymised results of routinely performed clinical tests reported in either clinical case notes or electronic medical records (EMR) were extracted from a web-based OpenClinica (OC) data capture system (Community edition). Information about participants' status was collected every six months (via National Health System Digital Spine portal or an equivalent local system). Multiple imputation by the chain equations method was used to impute missing data (mice v3.8.0 package R). High-throughput sequencing generated raw pair-end counts of 205,259 transcripts across 508 samples that belong to GenCode Release 28 (GRCh38.p12). Salmon (https://combine-lab.github.io/salmon/) was used to estimate the relative abundance of the transcripts (TPM, units of Transcripts Per Million) which were then mapped to genes (n = 60,144) using the tximport R package. TaqMan PCR results were captured and analysed using SDS version 2.4.
Data analysis	The code used to generate the results of this study is open-source and publicaly available at https://zenodo.org/badge/latestdoi/299615578.

For manuscripts utilizing custom algorithms or software that are central to the research but not yet described in published literature, software must be made available to editors and reviewers. We strongly encourage code deposition in a community repository (e.g. GitHub). See the Nature Research guidelines for submitting code & software for further information.

Data

Policy information about availability of data

All manuscripts must include a data availability statement. This statement should provide the following information, where applicable:
- Accession codes, unique identifiers, or web links for publicly available datasets
- A list of figures that have associated raw data
- A description of any restrictions on data availability

The transcriptomic and clinical data used in this study have been deposited in the EGA (the European Genome-phenome Archive) database under accession code EGAS00001005532 [https://ega-archive.org/studies/EGAS00001005532]. In compliance with the Ethics under which these data and samples have been collected,

the transcriptomic data are available through restricted access for approved researchers who agree to the conditions of use i.e. keeping it secure and only using it for approved purposes. To apply for access please contact cohortcoordination@medschl.cam.ac.uk. You will receive an application form within 30 days. The 'UK National PAH Cohort Study Data Access Committee' will review requests within three months of receipt of the completed application form and if approved, provide details for access to the RNAseq data stored at the EGA. All requesters must agree to the data access conditions found in EGA.
The data used to generate statistics, plots and figures are accessible through our interactive portal found in https://sheffield-university.shinyapps.io/ipah-rnaseq-app/. Source data are provided with this paper.

Field-specific reporting

Please select the one below that is the best fit for your research. If you are not sure, read the appropriate sections before making your selection.

- [x] Life sciences
- [ ] Behavioural & social sciences
- [ ] Ecological, evolutionary & environmental sciences

For a reference copy of the document with all sections, see nature.com/documents/nr-reporting-summary-flat.pdf

Life sciences study design

All studies must disclose on these points even when the disclosure is negative.

Sample size	Patients diagnosed with I/HPAH, PVOD or PCH, relatives of index cases and unrelated healthy controls were recruited at 9 UK centres between 14/01/2014 (date of the last sample profiled by RNASeq). With 359 samples, we have 99% power to detect 300 differentially expressed genes between subgroups at 5% FDR.
Data exclusions	359 patient samples used in this study was after some patients were excluded due to misdiagnoses as I/HPAH, and some RNA samples were excluded due to failing quality control. The clinical dataset was initially cleaned and filtered on 119 features that were identified by a domain expert from the original 887 features that described the dataset. Only genes with more than two reads (in a transcript level) in at least 95% of control and patient samples were considered and 11 additional male genes were removed (n = 25,955).
Replication	Five patients were sequenced twice across two sequencing runs to check that the expression profiles are reproducible, but only one replicate of each was used in the analysis. These replicate samples clustered together based on the principal components of their expression profiles. Clinical signatures of the RNAseq based subgroups were validated by the primary outcome (overall survival) in an additional cohort of 197 samples.
Randomization	Samples were assigned randomly to training and test sets for machine learning.
Blinding	All RNAseq samples and patients were anonymised.

Reporting for specific materials, systems and methods

We require information from authors about some types of materials, experimental systems and methods used in many studies. Here, indicate whether each material, system or method listed is relevant to your study. If you are not sure if a list item applies to your research, read the appropriate section before selecting a response.

Materials & experimental systems

n/a	Involved in the study	
x		Antibodies
x		Eukaryotic cell lines
x		Palaeontology and archaeology
x		Animals and other organisms
	x	Human research participants
	x	Clinical data
x		Dual use research of concern

Methods

n/a	Involved in the study	
x		ChIP-seq
x		Flow cytometry
x		MRI-based neuroimaging

Human research participants

Policy information about studies involving human research participants

Population characteristics	Clinical, functional and hemodynamic characteristics at the time of PAH diagnosis were prospectively entered into the database. The date of diagnosis corresponded to that of confirmatory right heart catheterisation. The average age in years of the cohort was 52 [42-64], age at diagnosis was 47 [35-59], 253 (70%) female, 23 (16.4%) vasoresponders, 387 [300-449] six minute walk distance in metres, and 222.5 [78.9-1162.8] NT-proBNP in ng/l.
Recruitment	Patients with idiopathic, heritable, or drug-induced pulmonary arterial hypertension (referred to throughout as PAH) were recruited from expert centers across the UK as part of the PAH Cohort study (www.ipahcohort.com). In each case, diagnosis was confirmed by right heart catheterization following established WHO international guidelines, which remained unchanged for the duration of this study. Healthy volunteers were recruited at the same centers and samples processed using the same standard operating procedure at all sites. All individuals gave written, informed consent with local ethical committee approval. Both prevalent and incident cases were allowed. Prevalent cases were defined as diagnosed earlier than six months before the study initiation. Patients in Cohort study were followed longitudinally as part of their clinical PAH care. All cases were diagnosed between March 1994 and November 2016, and diagnostic classification was made according to international guidelines. Patients with PAH associated with anorexigen exposure were considered as IPAH, whereas HPAH was defined by the presence of positive family history of PAH. In most centres, patients were seen every 3-6 months with an assessment of functional status and exercise capacity. Right heart catheterisation was repeated when considered necessary by the responsible clinician. Study visits were performed every 6 months. Healthy controls had been sampled only once and had clinical information recorded from the time of sampling. In total 358 patients, 13 relatives, 21 healthy controls recruited to the I/HPAH Cohort study were analysed.
Ethics oversight	UK National Cohort Study of Idiopathic and Heritable Pulmonary Arterial Hypertension (UK REC Ref 13/EE/0203) and/or the Sheffield Teaching Hospitals Observational Study of Pulmonary Hypertension, Cardiovascular and other Respiratory Disease (UK REC Ref 18/YH/0441).

Note that full information on the approval of the study protocol must also be provided in the manuscript.

Clinical data

Policy information about clinical studies

All manuscripts should comply with the ICMJEguidelines for publication of clinical research and a completedCONSORT checklist must be included with all submissions.

Clinical trial registration	NCT01907295
Study protocol	Observational study of 3600 participants with 5 year followup. Full study protocol can be accessed at https://www.ipahcohort.com/.
Data collection	Pseudonymised results of routinely performed clinical tests reported in either clinical case notes or electronic medical records (EMR) were stored in web-based OpenClinica (OC) data capture system (Community edition) hosted at the University of Cambridge. Twenty electronic Clinical Case Report Forms (eCRFs) distributed across seven events (Diagnostic, Continuous data, Follow-up, Epidemiology questionnaire, Suspension, Relatives, Unrelated healthy control) were constructed to accommodate routinely available clinical information. Details regarding data verification procedures are described in Swietlik, E. M. et al. Bayesian inference associates rare KDR variants with specific phenotypes in pulmonary arterial hypertension. Genetics 275 (2019).
Outcomes	Overall survival was the primary outcome measure for differences between RNA subgroups. Secondary outcome measures include REVEAL risk, changes in 6 minute walk distance in meters, admissions to hospital for PAH and cause of death. Incidence of new cases of PAH will be measured in relatives as well. Twenty electronic Clinical Case Report Forms (eCRFs) distributed across seven events (Diagnostic, Continuous data, Follow-up, Epidemiology questionnaire, Suspension, Relatives, Unrelated healthy control) were constructed to accommodate routinely available clinical information. Details regarding data verification procedures were previously described in detail. Information about participants' status was collected every six months (via National Health System Digital Spine portal or an equivalent local system). Current analysis was performed on the census performed on 31st January 2020. Two risk assessment strategies were applied to the data. Reveal risk score4 and abbreviated ERS risk scores were calculated in all patients who had the necessary minimum phenotypic information available. Patients who died or were transplanted were suspended on the day of the event, patients who withdrew from the study were censored on the date of the last visit, the reason for withdrawal was recorded.
